# Vitamin E Intake Attenuated the Association Between Elevated Blood Heavy Metal (Pb, Cd, and Hg) Concentrations and Diabetes Risk in Adults Aged 18–65 Years: Findings from 2007–2018 NHANES

**DOI:** 10.3390/toxics13010009

**Published:** 2024-12-25

**Authors:** Chenggang Yang, Shimiao Dai, Yutian Luo, Qingqing Lv, Junying Zhu, Aolin Yang, Zhan Shi, Ziyu Han, Ruirui Yu, Jialei Yang, Longjian Liu, Ji-Chang Zhou

**Affiliations:** 1 School of Public Health (Shenzhen), Shenzhen Campus of Sun Yat-sen University, Shenzhen 518107, China; yangchg5@mail2.sysu.edu.cn (C.Y.); daishm5@mail2.sysu.edu.cn (S.D.); lvqq6@mail2.sysu.edu.cn (Q.L.); zhujy85@mail2.sysu.edu.cn (J.Z.); yangaolin@mail2.sysu.edu.cn (A.Y.); shizh28@mail2.sysu.edu.cn (Z.S.); hanzy8@mail2.sysu.edu.cn (Z.H.); yurr3@mail2.sysu.edu.cn (R.Y.); yangjlei5@mail2.sysu.edu.cn (J.Y.); 2 School of Public Health, Columbia University, New York, NY 10025, USA; yl4608@caa.columbia.edu; 3 The Seventh Affiliated Hospital, Sun Yat-sen University, Shenzhen 518107, China; 4 Department of Epidemiology and Biostatistics, Dornsife School of Public Health, Drexel University, Philadelphia, PA 19104, USA; 5 Guangdong Province Engineering Laboratory for Nutrition Translation, Shenzhen 518107, China; 6 Guangdong Provincial Key Laboratory of Food, Nutrition and Health, Guangzhou 510080, China

**Keywords:** heavy metals, diabetes, glucose metabolic biomarkers, vitamin E, mortality, age-stratified analysis

## Abstract

The association between heavy metal exposure and diabetes is controversial and vitamin E (VE) may reduce diabetes risk. We aimed to examine the associations between blood heavy metals (BHMs) and diabetes risk and VE’s role in the relationship. From the 2007–2018 NHANES, 10,721 participants aged ≥ 18 were included for multiple statistical analyses, which revealed that BHMs and dietary VE intake were negatively associated with diabetes and fasting plasma glucose (FPG). The diabetes prevalence in each quartile (Q) of heavy metal exposure increased with age, but within age Q4, it generally decreased with exposure quartiles. Moreover, BHMs were positively associated with all-cause and diabetes-related mortalities with aging, which induced an age breakpoint of 65 years for age-stratified analyses on the associations between BHMs and diabetes risk. In those aged > 65, BHMs were negatively correlated with diabetes risk and its biomarkers; however, in adults aged 18–65, the correlation was positive. At higher VE intake levels, blood lead was associated with a lower diabetes risk and all three BHMs demonstrated lower FPG levels than those at lower VE intake levels. In conclusion, consuming sufficient VE and avoiding heavy metal exposure are highly recommended to reduce diabetes risk.

## 1. Introduction

Diabetes is one of the leading chronic non-communicable diseases [1]. In 2021, the global age-standardized prevalence of diabetes was estimated at 6.1% [2], with the number of people living with diabetes projected to reach 700 million by 2045 [3,4]. Given this significant public health burden, exploring prevention and treatment strategies for diabetes and its risk factors is crucial.

While excessive energy intake and insufficient physical activity (PA) are primary contributors to the increasing prevalence of diabetes [5,6], additional risk factors are emerging. Industrialization has escalated environmental pollution from heavy metals over decades, particularly in developing countries [7]. Numerous epidemiological studies and animal experiments, focusing on a single heavy metal exposure [8,9,10,11,12,13], have indicated that heavy metals can increase diabetes risk and fasting plasma glucose (FPG) levels. However, some reports suggest a negative or non-significant correlation between heavy metal exposure and diabetes risk [14,15,16,17].

On the other hand, some dietary nutrients play protective roles against the risks of diabetes. Vitamin E (VE) refers to a class of tocopherols and tocotrienols possessing antioxidant activity [18]. Supplementation with a high dose of VE, combined with vitamin A (VA) and zinc, may improve glycemic control, β-cell function, and insulin secretion in adults with type 2 diabetes [19], and the co-supplementation of VE and magnesium in women with gestational diabetes mellitus (GDM) can significantly improve glycemic control and lipid profiles [20].

Therefore, to explore the conflicting findings of the associations of blood heavy metals (BHMs) including lead (Pb), cadmium (Cd), and mercury (Hg) with diabetes risk and its related glucose metabolic biomarkers, and to examine the combined effect of BHMs and VE on diabetes risk, we included participants from the National Health and Nutrition Examination Survey (NHANES) with the relevant data, composing a study population.

## 2. Materials and Methods

### 2.1. Data Sources

NHANES, a cross-sectional population survey in the USA, received approval from the National Centers for Health Statics (NCHS) Institutional Review Board, and all participants provided informed consent (https://wwwn.cdc.gov/nchs/nhanes/default.aspx (accessed on 22 December 2024)). Our study selected data from the 2007–2018 cycles with information on demographics, diabetes, diet, and laboratory examination, along with information related to BHMs including Pb, Cd [21], and Hg [9]. Data from other cycles were not selected due to missing information on GDM, which was one of our exclusion criteria described below.

### 2.2. Exclusion Criteria

**Figure 1:**
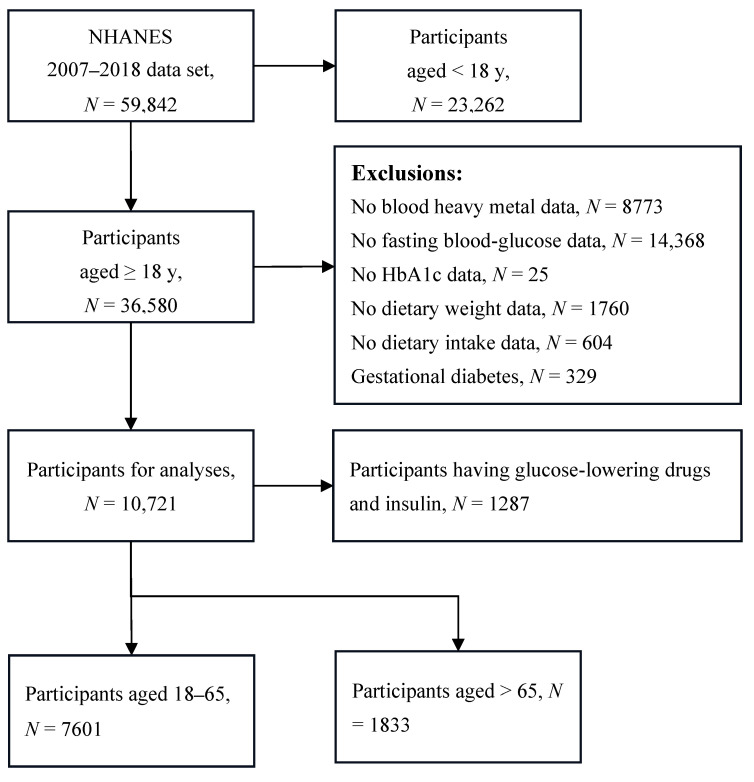
Flow chart of participant recruitment, NHANES 2007–2018.

Among the 59,842 participants, we excluded those aged less than 18 years (*N* = 23,262), those with incomplete surveys or missing values (*N* = 25,530), and those with GDM (*N* = 329). Then, we matched participants’ demographic and sociological information with data on dietary VE intake, diabetes, FPG, serum insulin, blood hemoglobin A1c (HbA1c), and BHMs, based on each participant’s code. Finally, we included 10,721 participants aged 18 and above for the analyses (Figure 1).

### 2.3. Measurement of BHM Levels

Pb, Cd, and Hg concentrations in whole blood were measured using inductively coupled plasma mass spectrometry, and the NHANES quality assurance and quality control (QA/QC) protocols meet the 1988 Clinical Laboratory Improvement Amendments mandates. Detailed QA/QC instructions are discussed in the NHANES Laboratory/Medical Technologists Procedures Manual (LPM) [22]. For biomarkers with concentrations below the detection limits, values were given as the detection limits divided by √2. The NHANES quality assurance and quality control protocols adhere to the 1988 Clinical Laboratory Improvement Amendments.

### 2.4. Definition of Diabetes and Insulin Resistance

Diabetes was defined by a self-reported doctor’s diagnosis of diabetes, using antidiabetic drugs (i.e., insulin and hypoglycemic agents), FPG levels ≥ 7.0 mmol/L (126 mg/dL), or blood HbA1c levels ≥ 6.5% (48 mmol/mol) [23]. The homeostatic model assessment for insulin resistance (HOMA-IR) = FPG level (mmol/L) × fasting serum insulin level (μU/mL)/22.5. Insulin resistance should be noted when HOMA-IR > 1.

### 2.5. Mortality Outcomes

To determine the mortality status in the follow-up population, we used the NHANES-linked mortality file as of 31 December 2019, which correlated with the National Death Index in the NCHS through a probability matching algorithm.

### 2.6. Dietary Nutrient Intakes

Dietary intakes of VE and other antioxidant nutrients like VA, vitamin C (VC), and selenium were assessed by 24 h food recall on two nonconsecutive days. Primary dietary recalls were conducted by trained interviewers at NHANES mobile examination centers. Dietary nutrient intake was calculated using the Food and Nutrient Database for Dietary Studies [24].

### 2.7. Statistical Analysis

Continuous variables were reported by means ± standard deviations, and categorical variables were expressed as *N* (%). Blood levels of Pb, Cd, and Hg were log-transformed, denoted by “Ln-Pb”, “Ln-Cd”, and “Ln-Hg”, respectively, to address distribution skewness.

Cox proportional hazards (PH) regression models were used to estimate hazard ratios (HRs) and 95% confidence intervals (95% CIs) of all-cause and diabetes-related mortalities associated with BHMs. We plotted cumulative Kaplan–Meier (K-M) curves for mortality during follow-up according to the levels of BHMs.

The segmented package for R software was used to analyze the age breakpoint in the relationships of age with diabetes prevalence and glucose metabolic biomarkers.

Logistic regression and multiple linear regression (MLR) analyses were utilized to evaluate the relationships of BHMs with diabetes and its biomarkers, respectively. Restricted cubic spline (RCS) models with four knots were employed in the regression analyses to explore the non-linear associations of BHMs with diabetes and its risk parameters of glucose, insulin, HOMA-IR, and HbA1c.

Weighted quantile sum (WQS) regression was conducted in conjunction with logistic regression for binary outcomes using the ‘g*WQS*’ package to evaluate the effects of mixed exposure to different types of BHMs [25]. The analysis provided a weighted linear index (WQS index) and the corresponding weight of each chemical, ranging from 0 to 1, indicating how much a specific pollutant contributed to the WQS index.

Results were presented as odds ratios (ORs) and 95% CIs for logistic regression while βs and 95% CIs were used for linear regression. Initially, the crude model without any adjustment was analyzed. Subsequently, Model 1 was adjusted for gender, age, and race. Model 2 included additional adjustments for educational level, the ratio of family income to poverty (RFIP), body mass index (BMI), smoking, and PA. Model 3 further incorporated adjustments for high cholesterol and hypertension. Finally, Model 4 extended adjustments to include the use of antidiabetic drugs. We calculated 12-year weighted values to account for the complex sampling design and weighting in all participants. All statistical analyses were performed with R 4.2.2. All reported probabilities (*p* values) were two-sided and *p* < 0.05 was considered statistically significant.

## 3. Results

### 3.1. Basic Characteristics of Participants

Among the 10,721 participants in the 2007–2018 NHANES dataset, 1921 (17.9%) were identified as having diabetes. Compared with participants without diabetes, those with diabetes had a higher age, men-to-women ratio, high BMI proportion, non-Hispanic black race proportion, and circulating levels of Pb, glucose, insulin, and HbA1c, along with higher HOMA-IR. Moreover, they had lower levels of education, PA, RFIP, serum cotinine, and blood Cd, as well as lower dietary intakes of VC, VE, and selenium (*p* < 0.05; Table 1).

Table S1 shows the BHM concentrations of each survey cycle. The blood Pb concentrations ranged from 1.2 to 1.8 μg/dL, showing a downward trend over time (*p* < 0.05), while the blood Cd concentrations ranged from 0.47 to 0.55 μg/L, and the blood Hg concentrations were from 1.3 to 1.6 μg/L.

### 3.2. Associations of Exposures with Diabetes and Its Biomarkers in All Participants

Tables S2–S6 present associations of BHMs and VE intake with diabetes, glucose, insulin, HOMA-IR, and HbA1c in all adult participants. In Models 1–3 of logistic regression analyses, the highest quartiles (Q4s) of the three BHM levels had a lower diabetes risk than the corresponding Q1 [in Model 3, ORs (95% CIs) = 0.31 (0.21, 0.45) for Ln-Pb, 0.68 (0.48, 0.97) for Ln-Cd, and 0.63 (0.45, 0.88) for Ln-Hg] (Table S2; *P*_trend_ < 0.05). However, after adjusting for the use of antidiabetic drugs in Model 4, the associations were no longer significant. In Models 1–4 of MLR analyses, Ln-Pb was negatively associated with glucose, insulin, HOMA-IR, and HbA1c levels (Tables S3–S6; *p* < 0.05), and Ln-Cd was negatively associated with insulin and HOMA-IR levels (Tables S4 and S5; *p* < 0.05). However, Ln-Hg was negatively associated with only HbA1c levels in multiple models (Models 1–3; *p* < 0.05; Table S6). For VE intake, Q4 had a lower risk of diabetes compared to Q1 in Models 1–3 [in Model 3, OR (95% CI) = 0.38 (0.21, 0.71)] Table S2; *P*_trend_ < 0.05), and the continuous variable of VE intake was negatively associated with glucose, insulin, and HOMA-IR in Models 1–4 (Tables S3–S5; *p* < 0.05).

In all participants, BHM levels were negatively associated with the risk of diabetes in Models 1–3, with Ln-Pb having the highest weight, followed by Ln-Cd (Figure S1A,B). BHM levels were also negatively correlated with blood glucose levels in Models 1–4, with Ln-Pb having the highest weight, followed by Ln-Hg (Figure S1C,D).

### 3.3. Effects of Age and Mortality on the Association Between BMHs and Diabetes Prevalence

The diabetes prevalence in all exposure quartiles increased with age (*p* < 0.05; Table 2). Within Q4 of age, the prevalence was the lowest in Q4 of exposure (*p* < 0.05). Thus, we suspected a significant increase in exposure-related mortality contributing to this phenomenon and analyzed the relationships of BHMs with overall mortality and diabetes-related mortality, as described below.

**Figure 2:**
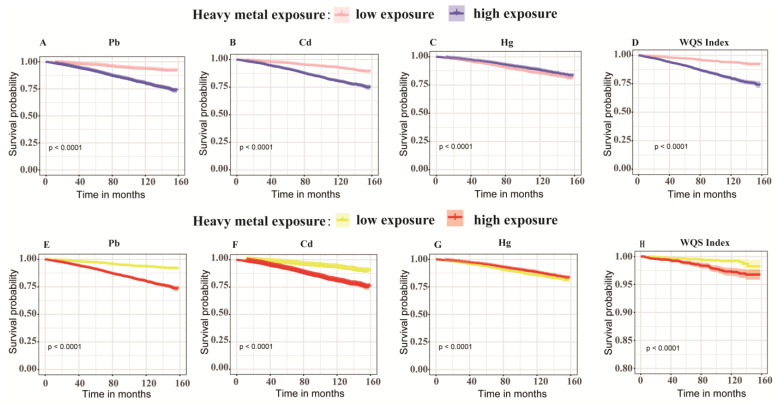
Kaplan–Meier survival curves depicting the unadjusted relationships of heavy metal exposure levels with all-cause (**A**–**D**) and diabetes-related mortalities (**E**–**H**). Data are population survival at specific time points. *N* = 10,721 in heavy metals analysis; *N* = 6464 in WQS analysis. Cd, cadmium; Hg, mercury; Pb, lead; WQS, weighted quantile sum.

**Figure 3:**
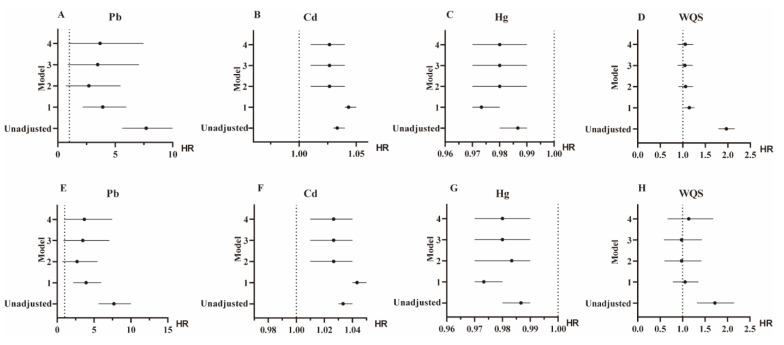
Cox regression analysis of heavy metals with overall (**A**–**D**) and diabetes-related mortalities (**E**–**H**). Data are hazard ratios and 95% confidence intervals. Model 1, adjusted for age, gender, and race. Model 2, adjusted for factors in Model 1 plus education, cotinine, body mass index, ratio of family income to poverty, and physical activity. Model 3, adjusted for factors in Model 2 plus hypertension and hypercholesteremia. Model 4, adjusted for factors in Model 3 plus medication histories of antidiabetic drugs and insulin. *N* = 10,721 in heavy metals analysis; *N* = 6464 in WQS analysis. Cd, cadmium; Hg, mercury; HR, hazard ratio; Pb, lead; WQS, weighted quantile sum.

In the death cohort analysis on the included 10,721 participants, the all-cause and diabetes-related mortalities were 10.0% and 8.8%, respectively. We divided participants into high and low-exposure subgroups by the medians of BHMs (Ln-Pb, Ln-Cd, and Ln-Hg) and WQS, and performed a survival analysis. Compared with the low exposure in the K-M survival curves (Figure 2), the high exposure to metals (Ln-Pb, Ln-Cd, and WQS) exhibited higher mortalities (*p* < 0.0001). The Cox regression analysis showed similar results to the K-M survival analysis. Ln-Pb in Model 1 and Ln-Cd in Models 1–4 were positively associated with overall and diabetes-related mortalities (Figure 3).

### 3.4. Age Breakpoint and Antidiabetic Drug Usage Being Considered for Further Analyses

As suggested by the above results of the negative associations between BHM levels and diabetes risk, as well as the changes in age- and BHM-associated diabetes prevalence and mortalities, we analyzed the age breakpoints in the relationships of age with diabetes prevalence and glucose metabolic biomarkers (Figure S2). Based on the result of age breakpoint analysis and the age defined as elderly, we selected 65 years as the breakpoint for age-stratified analyses. On the other hand, in Table S2, the Model 4 adjustment in the regression analysis suggested that the use of antidiabetic drugs was an important confounding factor affecting the association between BHMs and diabetes risk; so, we excluded those using antidiabetic drugs in the following brief analysis of those over 65 years and detailed analyses on participants aged 18–65.

### 3.5. Associations of Exposures with Diabetes and Its Biomarkers in Participants over 65 Years

In participants over 65 years (*N* = 1833), the exposure quantiles of blood Ln-Pb was negatively associated with the risk of diabetes in Models 1–3 (Table S7). For each unit increase in Ln-Pb, there was a 37.0% decrease in the risk of diabetes in Model 3 [OR (95% CI) = 0.63 (0.42, 0.93)]. For VE intake, Q4 [OR (95% CI) = 0.55 (0.31, 0.99)] and Q3 [OR (95% CI) = 0.44 (0.23, 0.81)] had lower risks of diabetes compared to Q1 in Model 3 (*P*_trend_ < 0.05).

The association between blood Ln-Pb and glucose was statistically negative (*p* < 0.05; Table S8), while the association between VE intake and glucose was not statistically significant.

### 3.6. Associations Between Exposures and Diabetes in Participants Aged 18–65

For the included 7601 adults aged 18–65, the age, gender, race, education, BMI, PA, glucose, insulin, HbA1c, HOMA-IR, and blood Ln-Pb were statistically different between diabetes and non-diabetes participants (*p* < 0.05; Table S9).

**Figure 4:**
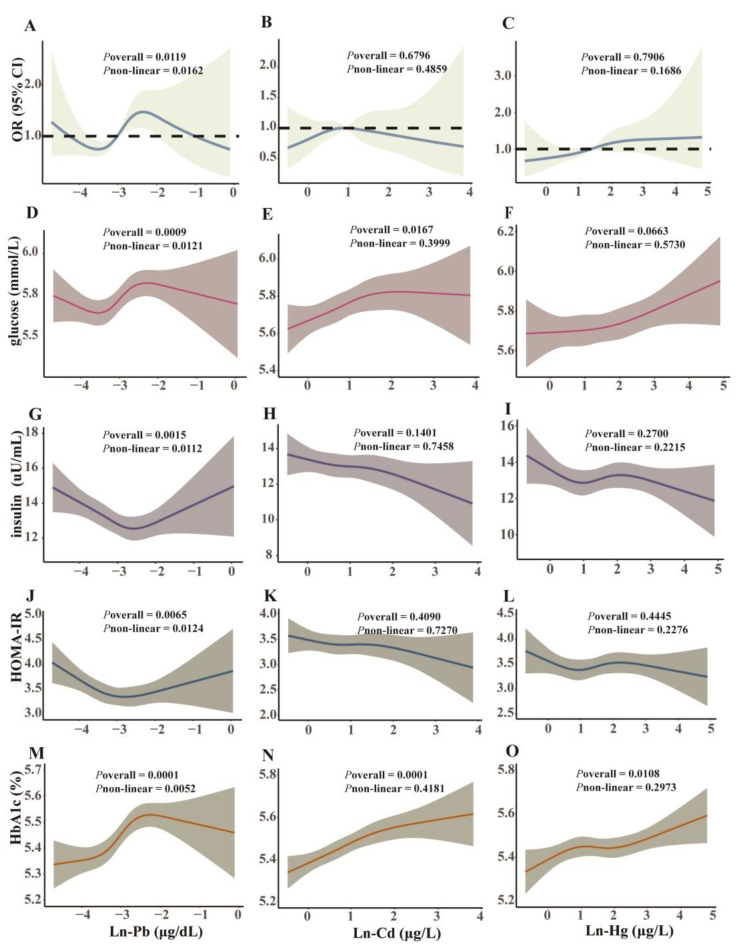
The odds ratio of diabetes associated with heavy metals (**A**–**C**) and the non-linear relationships of glucose metabolic biomarkers with heavy metals (**D**–**O**) in participants aged 18–65. Data are ORs (**A**–**C**) or estimated values (**D**–**O**) and 95% CIs. The model was adjusted for age, gender, race, education, cotinine, body mass index, ratio of family income to poverty, physical activity, hypertension, and hypercholesteremia. *N* = 7601. Cd, cadmium; CI, confidence interval; Hg, mercury; OR, odds ratio; Pb, lead.

Q4 [OR (95% CI) = 3.15 (1.67, 5.94)] and Q3 [OR (95% CI) = 1.86 (1.01, 3.40)] of blood Ln-Pb had higher risks of diabetes compared to Q1 in Model 2 (*P*_trend_ < 0.05); for each unit increase in Ln-Pb [OR (95% CI) = 1.24 (1.03, 1.49)], there was a 24% increase in the risk of diabetes in Model 1 (Table 3). A non-linear association between blood Ln-Pb and diabetes was observed (*P*_non-linear_ < 0.05), while no associations were found for other BHMs (Figure 4A–C).

The VE intake was not significantly associated with diabetes risk in the RCS model analysis (Figure S3A). However, VE intake was associated with a lower diabetes risk in the logistic regression analysis, and Q4 had a significantly lower risk compared to Q1 in Models 1–3 [OR (95% CI) = 0.27 (0.09, 0.77) in Model 3; *P*_trend_ = 0.01; Table 3]. For each unit increase in VE intake, there was a 9.0% decrease in the risk of diabetes in Model 3 [OR (95% CI) = 0.91 (0.84, 0.98); Table 3].

### 3.7. Associations Between Exposures and Glucose Metabolic Biomarkers in Participants Aged 18–65

In the survey-weighted MLR analysis, the associations of blood Ln-Pb, Ln-Cd (in Models 1–3), and Ln-Hg (in Models 2–3) with glucose (Table 4) and HbA1c (Table S10) were positive (*p* < 0.05). The high exposure quantiles (Q3s and Q4s) of blood Ln-Pb and Ln-Cd in Models 1–3 and Q4 of Ln-Hg in Model 2 had higher glucose and HbA1c levels compared to the corresponding Q1 (*P*_trend_ < 0.05). The associations of Ln-Pb and Ln-Cd in Models 1–3 and Ln-Hg in Model 1 with insulin levels (Table S11) and the associations of Ln-Pb, Ln-Cd, and Ln-Hg in Model 1 with HOMA-IR (Table S12) were significantly negative (*p* < 0.05). Meanwhile, the VE intake was negatively associated with glucose [β (95% CI) = −0.01 (−0.01, −0.00); Table 4] and HbA1c [β (95% CI) = −0.01 (−0.01, −0.01); Table S11] in Model 1, as well as with insulin and HOMA-IR in Models 1–3 [*p* < 0.05; Tables S12 and S13].

In the RCS model analysis (Figure 4D,O), blood Ln-Pb (*P*_non-linear_ < 0.05), but not Ln-Cd and Ln-Hg (*P*_non-linear_ > 0.05), was non-linearly associated with the four glucose metabolic biomarkers, i.e., glucose, insulin, HOMA-IR, and HbA1c. The associations of Ln-Pb with the four biomarkers, Ln-Cd with glucose and HbA1c, and Ln-Hg with HbA1c were positive (*P*_overall_ < 0.05). The VE intake was not non-linearly but negatively associated with glucose (*P*_overall_ = 0.026; Figure S3B).

**Figure 5:**
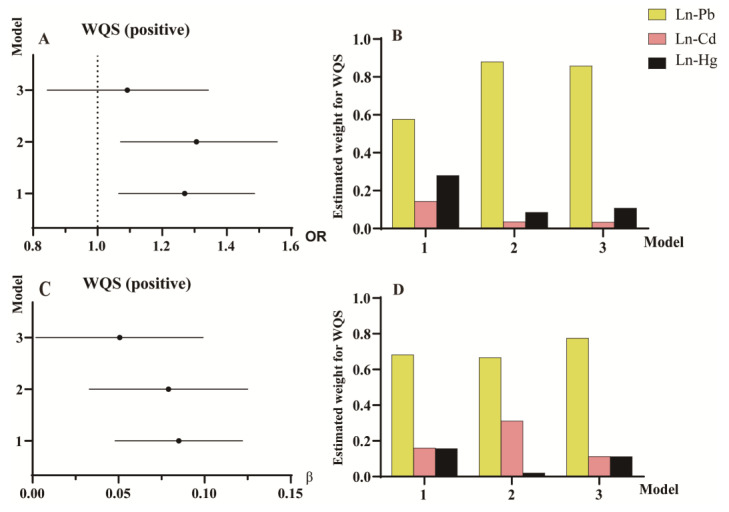
Estimated risk and weighted values of heavy metals for diabetes and glucose by WQS models in participants aged 18–65. (**A**) associations of blood heavy metals (BHMs) with diabetes risk (ORs and 95% CIs) in participants aged 18–65 years; (**B**) weighted values of BHMs for diabetes; (**C**) associations of BHMs with glucose (βs and 95% CIs) in participants aged 18–65 years; (**D**) weighted values of BHMs for glucose. Model 1, adjusted for age, gender, and race. Model 2, adjusted for factors in Model 1 plus education, cotinine, body mass index, ratio of family income to poverty, and physical activity. Model 3, adjusted for factors in Model 2 plus hypertension and hypercholesteremia. *N* = 7601. Cd, cadmium; CI, confidence interval; Hg, mercury; OR, odds ratio; Pb, lead; WQS, weighted quantile sum.

In the WQS regression analysis (Figure 5), BHM levels increased the risk of diabetes in Models 1–2 (Figure 5A,B), with Ln-Pb having the highest weight, followed by Ln-Hg. Moreover, there was a positive correlation between BHMs and blood glucose in Models 1–3 (Figure 5C,D), with Ln-Pb having the highest weight, followed by Ln-Cd. The results were consistent with those obtained from linear regression analysis.

### 3.8. Subgroup Analyses on the Associations of Exposures with Diabetes by Gender and BMI in Adults Aged 18–65

In the subgroup analysis by gender (Tables S13 and S14), the increased exposure to Ln-Pb, but not Ln-Cd and Ln-Hg, was associated with increased diabetes risks in both men and women. The blood Ln-Pb Q4 was associated with a higher diabetes risk compared to Q1 in Models 1–2 of men [OR (95% CI) = 2.18 (1.14, 4.19) in Model 2; *P*_trend_ < 0.05] and in Model 1 of women [OR (95% CI) = 1.88 (1.08, 3.27)]. For each unit increase in Ln-Pb, there was a 33% increase in the diabetes risk in Model 2 of men (*p* < 0.05). Regarding VE intakes, its Q4 had a decreased diabetes risk in Model 3 of women [OR (95% CI) = 0.12 (0.02, 0.74); *P*_trend_ = 0.03]. For each unit increase in VE, there was a 7.0% decrease in the diabetes risk in Model 1 of men and an 18.0% decrease in the diabetes risk in Model 3 of women (*p* < 0.05).

Tables S15 and S16 present the subgroup analysis by BMI with 25 kg/m2 as the cutoff value for normal weight and overweight/obese participants. In low BMI participants, Q4 of blood Ln-Pb was associated with a higher risk of diabetes compared to Q1 in Model 1 [OR (95% CI) = 2.99 (1.17, 9.28); *P*_trend_ < 0.01]. In high BMI participants, Q4 of blood Ln-Pb had a higher risk of diabetes compared to Q1 in Model 2 [OR (95% CI) = 2.07 (1.14, 3.75); *P*_trend_ = 0.04]. Moreover, for each unit increase in Ln-Pb, there was a 40% increase in the risk of diabetes in Model 1 in the low BMI participants (*p* < 0.05). Additionally, a continuous decrease of 8.0% in diabetes risk was observed for each unit increase in VE intake in the low BMI participants in Model 3 (*p* < 0.05).

### 3.9. Subgroup Analyses on the Associations of Exposures with Glucose by Gender and BMI in Adults Aged 18–65

Tables S17 and S18 present the subgroup analysis by gender. The associations of blood Ln-Pb in Models 1–2 and Ln-Hg in Model 2 with glucose were statistically positive in men. Q4 of blood Ln-Pb and Ln-Cd generally had higher glucose levels than the respective Q1 (*P*_trend_ < 0.05).

In women, the associations of the three BHMs in all the adjusted models, except Ln-Hg in Model 1 [β (95% CI) = 0.01 (−0.01, 0.04)], with glucose were statistically positive (*p* < 0.05). Q4s of blood Ln-Pb and Ln-Cd in Models 1–3 and Ln-Hg in Model 2 had higher glucose levels compared to the respective Q1 [βs (95% CIs) = 0.23 (0.12, 0.34) for Ln-Pb and 0.16 (0.02, 0.29) for Ln-Cd in Model 3 and 0.10 (0.02, 0.18) for Ln-Hg in Model 2; *P*_trend_ < 0.05]. Furthermore, the associations of VE intake with glucose in Models 1 and 3 of women were statistically significant.

Tables S19 and S20 present the subgroup analysis by BMI. In the low BMI participants, the associations of blood Ln-Pb with glucose in Models 1–2 were statistically positive (*p* < 0.05). Additionally, Q4 of blood Ln-Pb had higher glucose levels compared to Q1 in Models 1–2 [β (95% CI) = 0.18 (0.06, 0.29) in Model 2; *P*_trend_ < 0.01].

In the high BMI participants, the associations of blood Ln-Pb and Ln-Hg with glucose in Models 1–2 were statistically positive. Additionally, Q4s of blood Ln-Pb and Ln-Cd had higher glucose levels compared to the respective Q1 in Models 1–3 [βs (95% CIs) = 0.17 (0.04, 0.30) for Ln-Pb and 0.12 (0.01, 0.24) for Ln-Cd in Model 3; *P*_trend_ < 0.05].

### 3.10. Effects of VE Intake on the Associations of BHMs with Diabetes and Glucose

**Figure 6:**
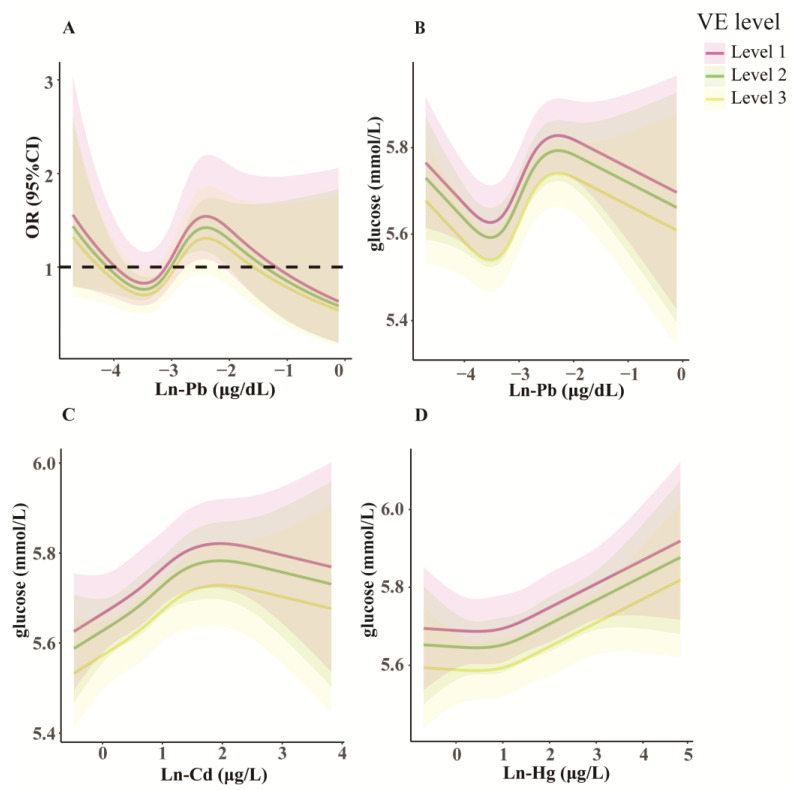
The associations of diabetes risk (**A**) and blood glucose levels (**B**–**D**) with heavy metals at different vitamin E (VE) levels. Data are ORs (**A**) or estimated values (**B**–**D**) and 95% CIs. The model was adjusted for age, gender, race, education, cotinine, body mass index, ratio of family income to poverty, physical activity, hypertension, and hypercholesteremia. *N* = 7601. Level 1, Quantile 1 of VE intake; Level 2, Quantiles 2–3 of VE intake; Level 3, Quantile 4 of VE intake. Cd, cadmium; CI, confidence interval; Hg, mercury; OR, odds ratio; Pb, lead.

We divided participants into three levels according to their VE intake quartiles, including Level 1 (Q1), Level 2 (Q2–3), and Level 3 (Q4). Figure 6A shows the RCS model revealing the non-linear relationships between Ln-Pb and diabetes risk across different VE levels. The diabetes risk was reduced with increasing levels of VE intake at the same Pb exposure level (Figure 6A). Additionally, positive correlations between BHM (particularly Pb) levels and glucose were noted across different VE levels, and glucose levels were reduced with increasing VE intake at the same exposure level of each BHM (Figure 6B–D).

Tables S21 and S22 show the relationships of BHM concentrations with glucose and diabetes at different VE intake levels in Models 2 and 3. In the two adjusted models at Levels 1–2 of VE intakes, diabetes risks in Q2 and Q4 of blood Ln-Pb were higher than those in Q1, and the glucose concentrations in Q3 and/or Q4 of blood Ln-Pb were higher than those in Q1 (*p* < 0.05). The glucose concentrations in Q3 of blood Ln-Hg at Level 2 of VE intake were higher than in Q1 (*p* < 0.05). In the two models at Level 3 of VE intake, the associations of blood Ln-Pb with diabetes and glucose become weaker or non-significant.

## 4. Discussion

Our study, via the baseline table analysis, discerned marked differences between diabetic and non-diabetic individuals. Elevated age, specific ethnicity, and a higher male ratio hinted at the combined influence of various factors in diabetes onset [26,27]. The increased BMI and blood Pb levels are linked to insulin resistance and glucose metabolism anomalies, aligning with past findings [28,29]. Diabetics’ lower education, physical activity, and nutrient (VC, VE, and selenium) intakes signify the role of lifestyle and nutrition [6,30,31,32]. The reduced blood Cd and serum cotinine might stem from metabolic changes and smoking habit distinctions, meriting further scrutiny to clarify their implications in diabetes.

Heavy metals represent significant risk factors for health. In recent years, researchers, investigating the association between heavy metal exposure and diabetes, found that BHMs and urine heavy metals were associated with an increased risk of diabetes in the NHANES survey of 2003–2014 [33], and a retrospective cohort study found that blood Pb concentrations at the beginning of the exposure might be an indicator of diabetes and glucose elevations [16]. However, a conflicting report found that higher levels of blood Pb, Cd, and Hg were associated with lower risks of metabolic syndrome in participants overall in the NHANES survey of 2011–2018 [14]. Utilizing NHANES data from 2007 to 2018, we also observed negative associations of BHMs with diabetes and glucose levels in adults of all ages (≥18 years old).

To explore the reasons for these contradictory reports, we suspected aging, mortality, and other confounding factors probably had strong effects. Then, we analyzed the changes in diabetes prevalence with aging as well as the relationships of BHM levels with overall and diabetes-related mortalities. As a result, we not only found that the increased diabetes prevalence at elder ages was decreased at higher BHM levels, but significant associations of high BHM levels with increased overall and diabetes-related mortalities were also revealed, and aging made the significance stronger. The increased mortality related to metal exposure was consistent with that reported by Duan et al. [34]. Furthermore, by examining the age breakpoints in the associations of age with diabetes and its biomarkers, we selected the age of 65 for the age-stratified analysis. Since the outcomes were highly variable with or without the use of antidiabetic drugs as a covariate adjustment (Tables S2 and S3), and the use of antidiabetic drugs also affects the authenticity of glucose metabolic indices, participants taking those drugs were excluded in the subsequent analyses.

Though the negative correlations of BHMs (especially Pb) with diabetes and glucose levels remained significant in participants over 65 years, they were reversed to positive correlations in participants aged 18–65 with multiple analysis methods, and Pb played a major role in the relationships. This age stratification provided a reasonable explanation for the previously conflicting reports on the associations of BHMs (especially Pb) with diabetes and its biomarkers [35]. Moreover, the relevant meta-analysis and epidemiological studies among the Chinese population all suggest that Pb exposure is closely related to an increased risk of diabetes [28,36]. Additionally, several in vivo studies conducted on animals have verified the correlation between Pb exposure and the progression of diabetes risk [37,38]. The underlying mechanism is that Pb might influence the overall glucose metabolism by inducing oxidative stress, which is thought to promote the diabetic state by directly affecting cellular signaling pathways that influence insulin signaling [39]. Pb also modifies intracellular signaling pathways to increase hepatic glucose production, which includes increased resting intracellular Ca^2+^, modulated protein kinase C activity, and a potential link between Pb and Rev-erb-α (NR1D1) [39,40]. In addition, heavy metals including Pb have deleterious effects on the metabolism of glucose and its association with other metabolic pathways, particularly glycolysis, glycogenesis, and gluconeogenesis, by altering and impairing the specific activities of major enzymes [41,42], eventually resulting in impaired FPG and hyperglycemia.

In the subgroup analysis, our results also showed that there were differences in the risk of diabetes associated with BHMs among populations with different characteristics. In particular, women seemed to be more susceptible to BHMs and an increased risk of hyperglycemia, consistent with the findings reported by Wang et al. [33], Seghieri et al. [43], and Hendryx et al. [44] Regarding BMI, individuals who were overweight/obese (BMI > 25 kg/m^2^) appeared to exhibit greater susceptibility to elevated BHMs, thereby escalating the risk of increased FPG levels. This phenomenon could be attributed to the potential exacerbation of glucose and lipid metabolism disorders in overweight/obese participants as a result of heavy metal exposure [45]. This finding is supported by the higher prevalence of cardiovascular diseases in obese patients [46]. We also found that smoking, low education level, low household income, hypertension, and hypercholesterolemia were associated with a higher risk of diabetes, which was consistent with many previous reports [47,48,49,50].

In addition to environmental pollution, lifestyle factors including VE intake are also important in the incidence of diabetes. Animal experiments have shown that VE could reverse the swelling of mitochondria permeability transition pores and increase mitochondrial lipid peroxidation levels in diabetic rats, thereby alleviating the apoptosis process [51]. Reno-Bernstein et al. [52] also found that compared with controls, VE-treated rats had threefold less cardiac oxidative stress, sixfold less mortality due to severe hypoglycemia, and sevenfold less incidence of heart block. Case–control studies have shown that VE supplementation has an important role in slowing down the progression of diabetic complications such as elevated post-prandial blood sugar, total cholesterol, and diastolic blood pressure [53]. Based on the evidence, we investigated the association between VE and diabetes and the interaction between VE and heavy metals on diabetes.

We categorized VE intake into different levels and found a negative association between VE intake and diabetes in the logistics regression, but it was not significant in the RCS model. This might be interpreted as follows: dietary nutrient intake needs to be further increased to show the antioxidant function [54]. Moreover, we analyzed the interaction of VE intake and blood Pb in their associations with diabetes risk and glucose levels. Compared with that in the low VE intake group, the association between blood Pb and glucose was attenuated in the high VE intake group, which was consistent with the findings of other studies [55]. One of the possible mechanisms is that VE improves oxidative stress and hepatocellular function. Al-Attar [56] investigated the effect of VE on heavy metal-induced diabetic nephropathy in rats and found that VE could alleviate oxidative stress and renal injury induced by heavy metals. These beneficial effects may also contribute to our observed improvements by increased VE intake on the associations of BHM levels with diabetes risk and its biomarkers, which, for the first time, indicated that VE may play a crucial role in mitigating the diabetes risk resulting from heavy metal exposure in humans, offering innovative approaches to diabetes prevention. A study has shown that the combined administration of zinc, selenium, and VE exhibited a more remarkable effect than either zinc or selenium and VE [57]; so, the combined administration of antioxidant micronutrients in the relationship between heavy metals and diabetes needs to be further explored. However, an epidemiological study has shown that a daily intake of VE of more than 400 IU increases the risk of death and should be avoided [58]; so, an appropriate supplementation of VE is needed to exert its antioxidant function.

## 5. Conclusions

In conclusion, our findings suggest that BHMs were positively associated with diabetes and blood glucose in those adults aged 18–65 years, which were attenuated by the increased VE intake. Moreover, the overweight/obese and female population were at a higher risk of diabetes in the presence of heavy metals exposure. The increased all-cause and diabetes-related mortalities associated with heavy metal exposures and aging enabled us to interpret the negative association between BHMs and diabetes risk in adults aged over 65 years and those of all ages. These results underscore the importance of continued efforts to control environmental pollution from heavy metals, strengthen the monitoring of human exposure, and improve VE intake to prevent adverse health effects. While this study provides valuable insights into the relationship between heavy metals and diabetes, as well as the role of VE intake in the relationship, prospective studies are also needed to overcome the limitations of the present study and establish a more robust understanding of these associations.

## Figures and Tables

**Table 1:** Basic characteristics of participants by diabetes, NHANES 2007–2018.

	All	No-Diabetes	Diabetes	*p* *
*N*	10,721	8800	1921	
Age, y, means ± SD	48.98 ± 18.59	46.17 ± 18.35	61.85 ± 13.52	<0.01
Gender, *n* (%)				< 0.01
male	5272 (49.2)	4189 (47.6)	1083 (56.4)	
female	5449 (50.8)	4611 (52.4)	838 (43.6)	
BMI, *n* (%)				<0.01
BMI < 25 kg/m^2^	3144 (29.7)	2882 (33.1)	262 (13.9)	
25 ≤ BMI < 30 kg/m^2^	3491 (32.9)	2955 (33.9)	536 (28.5)	
BMI ≥ 30 kg/m^2^	3965 (37.4)	2879 (33.0)	1086 (57.6)	
Race, *n* (%)				<0.01
Mexican American	1617 (15.1)	1300 (14.8)	317 (16.5)	
other Hispanic	1146 (10.7)	940 (10.7)	206 (10.7)	
non-Hispanic white	4655 (43.4)	3905 (44.4)	750 (39.0)	
non-Hispanic black	2197 (20.5)	1725 (19.6)	472 (24.6)	
other races	1106 (10.3)	930 (10.6)	176 (9.2)	
Education, *n* (%)				<0.01
<9th grade	1001 (9.3)	702 (8.0)	299 (15.6)	
9–11th grade	1402 (13.1)	1091 (12.4)	311 (16.2)	
high school graduate	2521 (23.5)	2040 (23.2)	481 (25.1)	
college level graduate	3141 (29.3)	2650 (30.1)	491 (25.6)	
≥college graduate	2644 (24.7)	2309 (26.3)	335 (17.5)	
RFIP	2.48 (1.62)	2.51 (1.63)	2.36 (1.53)	<0.01
Physical activity, *n* (%)				<0.01
high	3503 (43.8)	3124 (45.7)	379 (32.9)	
middle	4460 (55.8)	3693 (54.0)	767 (66.5)	
low	29 (0.4)	22 (0.3)	7 (0.6)	
Fasting circulating parameters, means ± SD				
serum cotinine, ng/mL	52.61 ± 123.23	53.77 ± 122.25	47.24 ± 127.58	0.04
blood Pb, μg/dL	1.51 ± 1.57	1.49 ± 1.59	1.63 ± 1.50	<0.01
blood Cd, μg/L	0.50 ± 0.57	0.51 ± 0.58	0.48 ± 0.52	0.04
blood Hg, μg/L	1.47 ± 2.20	1.47 ± 2.17	1.44 ± 2.32	0.56
plasma glucose, mmol/L	6.03 ± 1.88	5.48 ± 0.56	8.53 ± 3.27	<0.01
serum insulin, μU/mL	13.90 ± 16.23	12.36 ± 10.37	21.01 ± 30.36	<0.01
HOMA-IR	4.00 ± 6.32	3.08 ± 2.76	8.22 ± 12.95	<0.01
blood HbA1c, %	5.74 ± 1.02	5.43 ± 0.39	7.16 ± 1.63	<0.01
Nutrient intakes, means ± SD				
vitamin A, μg/day	607.42 ± 524.25	611.61 ± 539.04	588.20 ± 449.96	0.12
vitamin C, mg/day	83.78 ± 78.08	85.33 ± 80.12	76.68 ± 67.52	<0.01
vitamin E, mg/day	8.43 ± 6.40	8.52 ± 6.40	8.01 ± 6.35	0.01
selenium, μg/day	110.68 ± 52.52	111.60 ± 52.86	106.46 ± 50.74	<0.01

Note: BMI, body mass index; Cd, cadmium; FPG, fasting plasma glucose; HbA1c, hemoglobin A1c; Hg, mercury; HOMA-IR, homeostatic model assessment for insulin resistance; PA, physical activity; Pb, lead; RFIP, the ratio of family income to poverty; SD, standard deviations. * Between diabetes and non-diabetes.

**Table 2:** Prevalence of diabetes (%) in different ages at different exposure levels of heavy metals.

	Age				*p*
	Q1	Q2	Q3	Q4	
WQS					
Q1	2.35	4.24	10.12	37.53	<0.05
Q2	2.81	4.81	24.34	41.96	<0.05
Q3	4.33	18.50	30.86	30.12	<0.05
Q4	8.10	16.70	28.20	27.30	<0.05
Pb					
Q1	2.39	4.68	10.19	37.17	<0.05
Q2	2.28	4.77	24.10	42.73	<0.05
Q3	4.43	18.91	30.04	29.56	<0.05
Q4	8.42	14.81	28.54	27.23	<0.05
**Cd**					
Q1	3.41	4.37	21.11	40.99	<0.05
Q2	2.71	10.44	25.54	36.30	<0.05
Q3	3.71	11.65	27.48	33.18	<0.05
Q4	3.71	16.14	23.00	29.44	<0.05
Hg					
Q1	2.54	7.98	24.40	36.06	<0.05
Q2	4.77	9.56	24.87	38.61	<0.05
Q3	3.33	14.83	24.88	33.41	<0.05
Q4	3.17	13.67	24.82	28.51	<0.05

Note: Cd, cadmium; Hg, mercury; Pb, lead; Q1–4, Quartile 1–4; WQS, weighted quantile sum. *N* = 10,721.

**Table 3:** Adjusted odds ratios of diabetes associated with heavy metal exposures and VE intake in participants aged 18–65.

		Continuous	Q1	Q2	Q3	Q4	*P*_trend_
		OR (95% CI)	(Reference)	OR (95% CI)	OR (95% CI)	OR (95% CI)	
Ln concentrations of blood Pb (μg/dL), Cd, and Hg (μg/L)							
Ln-Pb	Model 1	1.24 (1.03, 1.49) *	1.00	1.34 (0.87, 2.8)	1.50 (1.01, 2.24) *	2.09 (1.41, 3.08) *	<0.01
	Model 2	1.30 (0.99, 1.72)	1.00	1.94 (1.06, 3.54) *	1.86 (1.01, 3.40) *	3.15 (1.67, 5.94) *	0.01
	Model 3	1.16 (0.82, 1.65)	1.00	1.69 (0.90, 3.161)	1.33 (0.67, 2.66)	2.40 (1.16, 4.95) *	0.07
Ln-Cd	Model 1	1.15 (0.98, 1.35)	1.00	1.37 (0.87, 2.18)	1.50 (0.98, 2.30)	1.34 (0.91, 1.98)	0.09
	Model 2	1.32 (0.99, 1.75)	1.00	1.63 (0.90, 2.95)	1.71 (1.03, 2.84) *	1.64 (0.89, 3.04)	0.05
	Model 3	1.24 (0.91, 1.69)	1.00	1.74 (0.94, 3.23)	1.63 (0.93, 2.83)	1.46 (0.74, 2.89)	0.15
Ln-Hg	Model 1	1.12 (0.97, 1.28)	1.00	1.01 (0.69, 1.48)	1.28 (0.83, 1.97)	1.31 (0.87, 1.97)	0.13
	Model 2	1.19 (0.99, 1.43)	1.00	0.97 (0.59, 1.58)	1.34 (0.75, 2.40)	1.41 (0.80, 2.48)	0.15
	Model 3	1.13 (0.92, 1.40)	1.00	0.97 (0.57, 1.64)	1.34 (0.71, 2.51)	1.19 (0.63, 2.25)	0.42
Intake, mg/day							
VE	Model 1	0.94 (0.89, 0.99) *	1.00	1.14 (0.44, 2.96)	0.67 (0.29, 1.54)	0.40 (0.18, 0.89) *	0.01
	Model 2	0.92 (0.85, 0.99) *	1.00	1.28 (0.43, 3.84)	0.53 (0.18, 1.59)	0.34 (0.12, 0.97) *	0.01
	Model 3	0.91 (0.84, 0.98) *	1.00	1.26 (0.39, 4.09)	0.46 (0.15, 1.41)	0.27 (0.09, 0.77) *	0.01

Note: Cd, cadmium; CI, confidence interval; Hg, mercury; OR, odds ratio; Pb, lead; Q1, Quartile 1 (as the reference group); Q2–4, Quartile 2–4; VE, vitamin E. Model 1, adjusted for age, gender, and race; Model 2, adjusted for factors in Model 1 plus education, cotinine, body mass index, ratio of family income to poverty, and physical activity; Model 3, adjusted for factors in Model 2 plus hypertension and hypercholesteremia. *N* = 7601. * *p* < 0.05.

**Table 4:** Multiple-adjusted linear regressions of glucose with heavy metal exposures and VE intake in participants aged 18–65.

		Continuous	Q1	Q2	Q3	Q4	*P*_trend_
		OR (95% CI)	(Reference)	OR (95% CI)	OR (95% CI)	OR (95% CI)	
Ln concentrations of blood Pb (μg/dL), Cd, and Hg (μg/L)							
Ln-Pb	Model 1	0.10 (0.05, 0.14) *	1.00	0.04 (−0.02, 0.10)	0.12 (0.06, 0.18) *	0.23 (0.15, 0.30) *	<0.01
	Model 2	0.10 (0.05, 0.14) *	1.00	0.07 (0.01, 0.14) *	0.14 (0.07, 0.20) *	0.25 (0.17, 0.34) *	<0.01
	Model 3	0.07 (0.01, 0.12) *	1.00	0.06 (−0.01, 0.13)	0.09 (0.02, 0.17) *	0.20 (0.11, 0.30) *	<0.01
Ln-Cd	Model 1	0.03 (0.01, 0.06) *	1.00	0.06 (−0.01, 0.13)	0.08 (0.02, 0.14) *	0.08 (0.02, 0.13) *	<0.01
	Model 2	0.06 (0.02, 0.11) *	1.00	0.08 (0.01, 0.14)	0.09 (0.03, 0.15) *	0.12 (0.04, 0.21) *	<0.01
	Model 3	0.06 (0.01, 0.11) *	1.00	0.11 (0.04, 0.17)	0.09 (0.03, 0.16) *	0.12 (0.03, 0.22) *	<0.01
Ln-Hg	Model 1	0.02 (−0.01, 0.04)	1.00	−0.01 (−0.07, 0.06)	0.01 (−0.06, 0.07)	0.03 (−0.03, 0.09)	0.30
	Model 2	0.04 (0.018, 0.07) *	1.00	−0.02 (−0.09, 0.05)	−0.01 (−0.08, 0.07)	0.08 (0.01, 0.15) *	0.03
	Model 3	0.04 (0.01, 0.06) *	1.00	−0.03 (−0.11, 0.05)	−0.01 (−0.10, 0.08)	0.04 (−0.04, 0.12)	0.26
Intake, mg/day							
VE	Model 1	−0.01 (−0.01, −0.00) *	1.00	−0.03 (−0.17, 0.12)	0.06 (−0.06, 0.17)	−0.07 (−0.17, 0.03)	0.28
	Model 2	−0.01 (−0.01, 0.01)	1.00	0.01 (−0.13, 0.15)	0.08 (−0.04, 0.20)	0.01 (−0.10, 0.11)	0.88
	Model 3	−0.01 (−0.01, 0.00)	1.00	−0.03 (−0.19, 0.14)	0.02 (−0.11, 0.15)	−0.08 (−0.20, 0.05)	0.24

Note: Cd, cadmium; CI, confidence interval; Hg, mercury; Pb, lead; Q1, Quartile 1 (as the reference group); Q2–4, Quartile 2–4; VE, vitamin E. Model 1, adjusted for age, gender, and race; Model 2, adjusted for factors in Model 1 plus education, cotinine, body mass index, ratio of family income to poverty, and physical activity; Model 3, adjusted for factors in Model 2 plus hypertension and hypercholesteremia. *N* = 7601. * *p* < 0.05.

## Data Availability

The data used in this study can be downloaded for free in NHANES (https://www.cdc.gov/nchs/nhanes/index.htm (accessed on 22 December 2024)). The code will be provided as required.

## Supplementary Materials

The following supporting information can be downloaded at: https://www.mdpi.com/article/10.3390/toxics13010009/s1, Table S1. Concentrations of blood heavy metals in the study participants, NHANES 2007–2018; Table S2. Adjusted odds ratios of diabetes associated with heavy metal exposures and VE intake in all adult participants; Table S3. Multiple-adjusted linear regressions of glucose with heavy metal exposure and VE intake in all adult participants; Table S4. Multiple-adjusted linear regressions of insulin with heavy metal exposures and VE intake in all adult participants; Table S5. Multiple-adjusted linear regressions of HOMA-IR with heavy metal exposures and VE intake in all adult participants; Table S6. Multiple-adjusted linear regressions of HbA1c with heavy metal exposures and VE intake in all adult participants; Table S7. Adjusted odds ratios of diabetes associated with heavy metal exposures and VE intake in participants aged > 65; Table S8. Multiple-adjusted linear regressions of glucose with heavy metal exposures and VE intake in participants aged > 65; Table S9. Basic profiles of participants aged 18–65 by diabetes, NHANES 2007–2018; Table S10. Multiple-adjusted linear regressions of HbA1c with heavy metal exposures and VE intake in participants aged 18–65; Table S11. Multiple-adjusted linear regressions of insulin with heavy metal exposures and VE intake in participants aged 18–65; Table S12. Multiple-adjusted linear regressions of HOMA-IR with heavy metal exposures and VE intake in participants aged 18–65; Table S13. Logistic regression between heavy metals and diabetes in men aged 18–65; Table S14. Logistic regression between heavy metals and diabetes in women aged 18–65; Table S15. Logistic regression between heavy metals and risk of diabetes in participants aged 18–65 with BMI < 25 kg/m^2^; Table S16. Logistic regression between heavy metals and risk of diabetes in participants aged 18–65 with BMI ≥ 25 kg/m^2^; Table S17. Multiple-adjusted linear regression between heavy metals and glucose in men aged 18–65; Table S18. Multiple-adjusted linear regression between heavy metals and glucose in women aged 18–65; Table S19. Multiple-adjusted linear regression between heavy metals and glucose in participants aged 18–65 with BMI < 25 kg/m^2^; Table S20. Multiple-adjusted linear regression between heavy metals and glucose in participants aged 18–65 with BMI ≥ 25 kg/m^2^; Table S21. Associations of blood heavy metals with diabetes and glucose by VE intake levels with Model 2 adjustment in participants aged 18–65; Table S22. Flow chart of participant recruitment, NHANES 2007–2018; Figure S1. Associations of metal co-exposure with diabetes (A, B) and blood glucose levels (C, D); Figure S2. Age breakpoints in the relationships of age with diabetes and its biomarkers; Figure S3. Odds ratio of diabetes with VE (A) and non-linear relationship of glucose with VE (B) in participants aged 18–65.
