# Supplementary material for: Vitamin E Intake Attenuated the Association Between Elevated Blood Heavy Metal (Pb, Cd, and Hg) Concentrations and Diabetes Risk in Adults Aged 18–65 Years: Findings from 2007–2018 NHANES
Source: Toxics. 2024 Dec 25;13(1):9. doi: 10.3390/toxics13010009 (PMC11769426; doi:10.3390/toxics13010009)
Supplement: Supplementary file 1 [file toxics-13-00009-s001.zip › toxics-3365411-supplementary.pdf]

Supplementary material

Supplementary figures (3) and tables (22):

Figure S1. Associations of metal co-exposure with diabetes and blood glucose levels.
Figure S2. Age breakpoints in the relationships of age with diabetes and its biomarkers.
Figure S3. Odds ratio of diabetes with VE and non-linear relationship of glucose with VE in participants aged 18 - 65.
Table S1. Concentrations of blood heavy metals in the study participants, NHANES 2007 - 2018.
Table S2. Adjusted odds ratios of diabetes associated with heavy metal exposures and VE intake in all adult participants.
Table S3. Multiple-adjusted linear regressions of glucose with heavy metal exposure and VE intake in all adult participants.
Table S4. Multiple-adjusted linear regressions of insulin with heavy metal exposures and VE intake in all adult participants.
Table S5. Multiple-adjusted linear regressions of HOMA-IR with heavy metal exposures and VE intake in all adult participants.
Table S6. Multiple-adjusted linear regressions of HbA1c with heavy metal exposures and VE intake in all adult participants.
Table S7. Adjusted odds ratios of diabetes associated with heavy metal exposures and VE intake in participants aged > 65.
Table S8. Multiple-adjusted linear regressions of glucose with heavy metal exposures and VE intake in participants aged > 65.
Table S9. Basic profiles of participants aged 18 - 65 by diabetes, NHANES 2007 - 2018.
Table S10. Multiple-adjusted linear regressions of HbA1c with heavy metal exposures and VE intake in participants aged 18 - 65.
Table S11. Multiple-adjusted linear regressions of insulin with heavy metal exposures and VE intake in participants aged 18 - 65.
Table S12. Multiple-adjusted linear regressions of HOMA-IR with heavy metal exposures and VE intake in participants aged 18 - 65.
Table S13. Logistic regression between heavy metals and diabetes in men aged 18 - 65.
Table S14. Logistic regression between heavy metals and diabetes in women aged 18 - 65.
Table S15. Logistic regression between heavy metals and risk of diabetes in participants aged 18 - 65 with BMI < 25 kg/m^2.
Table S16. Logistic regression between heavy metals and risk of diabetes in participants aged 18 - 65 with BMI ≥ 25 kg/m^2.
Table S17. Multiple-adjusted linear regression between heavy metals and glucose in men aged 18 - 65.
Table S18. Multiple-adjusted linear regression between heavy metals and glucose in women aged 18 - 65.
Table S19. Multiple-adjusted linear regression between heavy metals and glucose in participants aged 18 - 65 with BMI < 25 kg/m^2.

Table S20. Multiple-adjusted linear regression between heavy metals and glucose in participants aged 18 - 65 with BMI ≥ 25 kg/m^2.
Table S21. Associations of blood heavy metals with diabetes and glucose by VE intake levels with Model 2 adjustment in participants aged 18 - 65.
Table S22. Associations of blood heavy metals with diabetes and glucose by VE intake levels with Model 3 adjustment in participants aged 18 - 65.

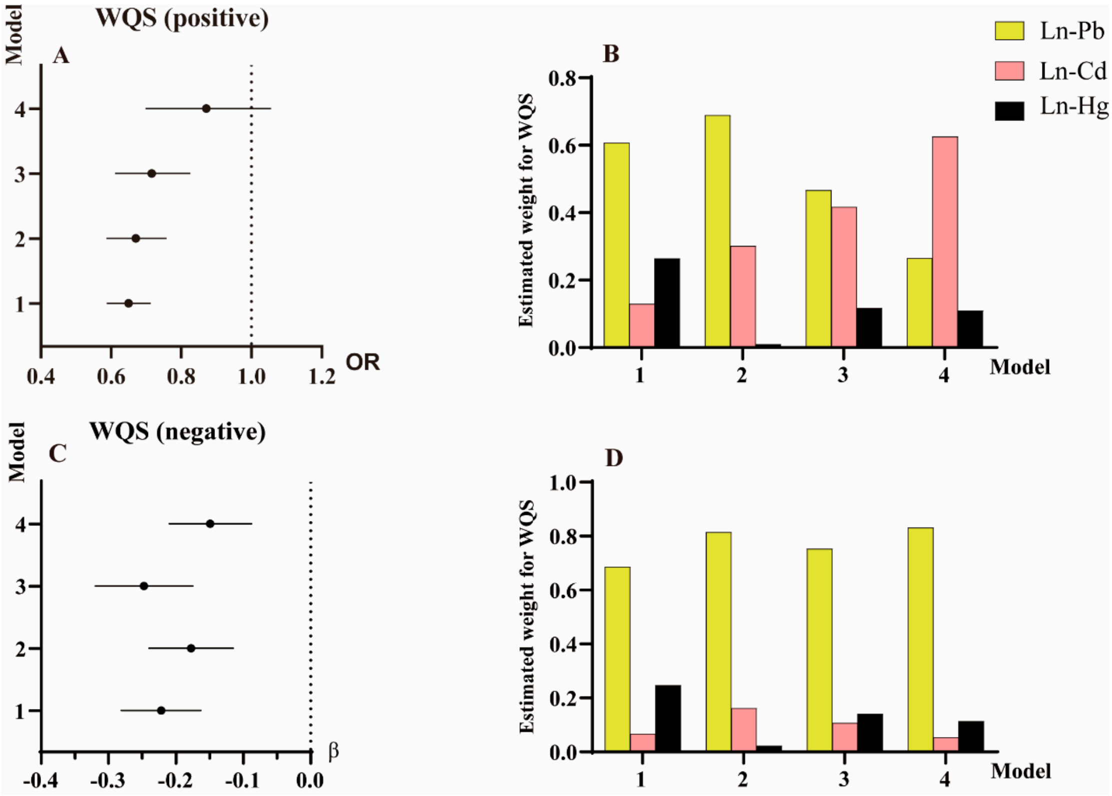

Figure S1. Associations of metal co-exposure with diabetes (A, B) and blood glucose levels (C, D). A, associations of blood heavy metals (BHMs) with diabetes; B, weighted values of BHMs for diabetes; C, associations of BHMs with glucose; D, weighted values of BHMs for glucose. Model 1, adjusted for age, gender, and race. Model 2, adjusted for factors in Model 1 plus education, cotinine, body mass index, ratio of family income to poverty, and physical activity. Model 3, adjusted for factors in Model 2 plus hypertension and hypercholesteremia. Model 4, adjusted for factors in Model 3 plus medication histories of antidiabetic drugs and insulin. N = 10,721. Cd, cadmium; Hg, mercury; OR, odds ratio; Pb, lead. WQS, weighted quantile sum.

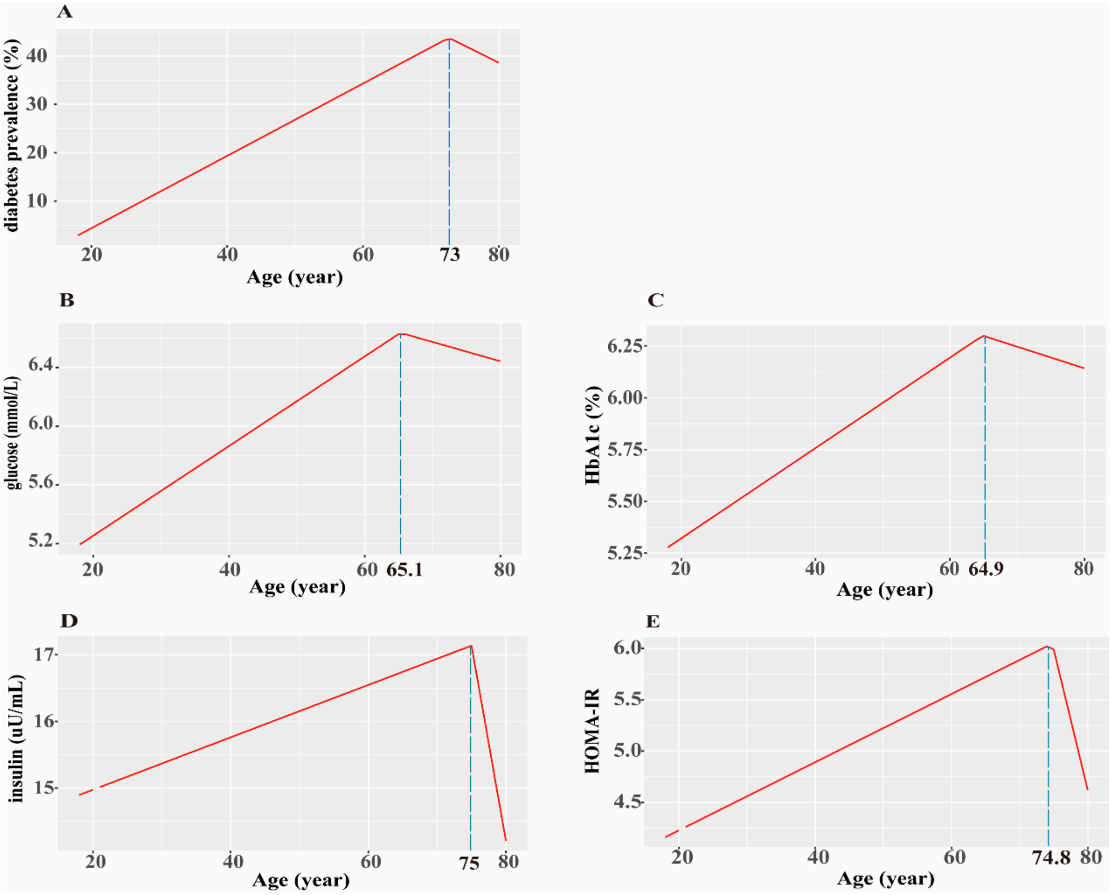

Figure S2. Age breakpoints in the relationships of age with diabetes and its biomarkers. Adjusted for gender and race. $N = 10{,}721$.

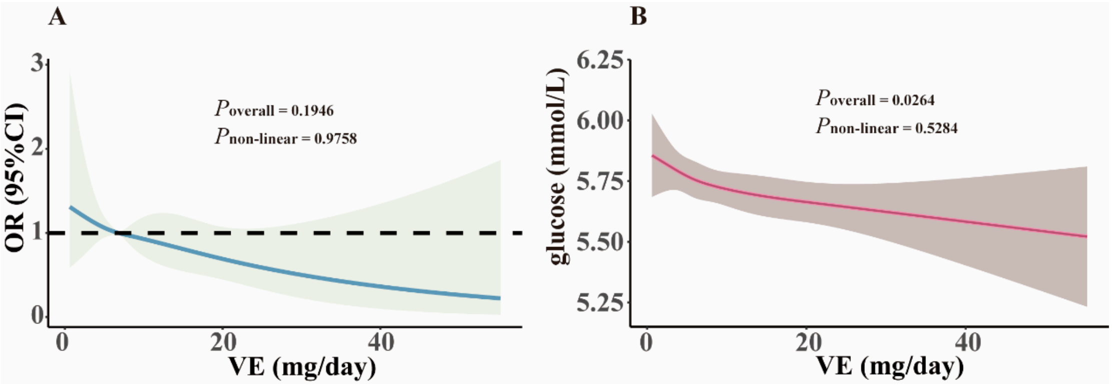

Figure S3. Odds ratio of diabetes with VE (A) and non-linear relationship of glucose with VE (B) in participants aged 18 - 65. Data are ORs and 95% CIs (A) or estimated values and 95% CIs (B). The model was adjusted for age, gender, race, education, cotinine, body mass index, ratio of family income to poverty, physical activity, hypertension, and hypercholesteremia. N = 7,601. CI, confidence interval; OR, odds ratio; VE, vitamin

Table S1. Concentrations (mean ± SD) of blood heavy metals in the study participants, NHANES 2007 - 2018.

Year	Lead (μg/dL)	Cadmium (μg/L)	Mercury (μg/L)
2007 - 2008	1.80 ± 1.65	0.55 ± 0.63	1.39 ± 1.92
2009 - 2010	1.63 ± 1.67	0.48 ± 0.48	1.60 ± 2.43
2011 - 2012	1.50 ± 1.91	0.53 ± 0.63	1.53 ± 2.12
2013 -2014	1.28 ± 1.16	0.47 ± 0.55	1.41 ± 2.11
2015 - 2016	1.27 ± 1.22	0.48 ± 0.54	1.37 ± 1.99
2017 - 2018	1.20 ± 1.19	0.48 ± 0.57	1.30 ± 2.31
P value	< 0.05	< 0.05	< 0.05

Note: SD, standard deviations. $N = 10,721$.

Table S2. Adjusted odds ratios of diabetes associated with heavy metal exposures and VE intake in all adult participants.

		Continuous OR (95% CI)	Q1 (Reference)	Q2 OR (95% CI)	Q3 OR (95% CI)	Q4 OR (95% CI)	P_{trend}
Ln concentrations of blood Pb (μg/dL), Cd, and Hg (μg/L)							
Ln-Pb	Model 1	0.50 (0.44, 0.57) *	1.00	0.60 (0.49, 0.73) *	0.42 (0.33, 0.53) *	0.31 (0.24, 0.40) *	< 0.01
	Model 2	0.46 (0.39, 0.55) *	1.00	0.65 (0.49, 0.86) *	0.38 (0.28, 0.51) *	0.32 (0.23, 0.46) *	< 0.01
	Model 3	0.45 (0.37, 0.54) *	1.00	0.63 (0.47, 0.83) *	0.36 (0.27, 0.49) *	0.31 (0.21, 0.45) *	< 0.01
	Model 4	0.73 (0.53, 1.01)	1.00	0.88 (0.52, 1.50)	0.61 (0.35, 1.08)	0.80 (0.45, 1.44)	0.34
Ln-Cd	Model 1	0.79 (0.69, 0.89) *	1.00	0.71 (0.56, 0.90) *	0.60 (0.47, 0.76) *	0.59 (0.47, 0.76) *	< 0.01
	Model 2	0.81 (0.67, 0.97) *	1.00	0.75 (0.56, 0.99)	0.63 (0.47, 0.84) *	0.67 (0.48, 0.93) *	< 0.01
	Model 3	0.81 (0.67, 0.98) *	1.00	0.78 (0.59, 1.05)	0.65 (0.49, 0.88) *	0.68 (0.48, 0.97) *	< 0.01
	Model 4	0.91 (0.69, 1.21)	1.00	0.80 (0.50, 1.28)	0.72 (0.44, 1.16)	0.88 (0.48, 1.42)	0.34
Ln-Hg	Model 1	0.86 (0.79, 0.92) *	1.00	0.92 (0.75, 1.14)	0.81 (0.65, 1.01)	0.68 (0.54, 0.85) *	< 0.01
	Model 2	0.89 (0.79, 1.00) *	1.00	0.80 (0.61, 1.05)	0.86 (0.65, 1.15)	0.68 (0.49, 0.94) *	0.05
	Model 3	0.87 (0.77, 0.98) *	1.00	0.81 (0.61, 1.06)	0.85 (0.62, 1.16)	0.63 (0.45, 0.88) *	0.02
	Model 4	1.07 (0.90, 1.28)	1.00	0.80 (0.54, 1.19)	1.17 (0.73, 1.87)	1.00 (0.60, 1.68)	0.63
Intake, mg/day							
VE	Model 1	0.98 (0.94, 1.01)	1.00	0.60 (0.34, 1.04)	0.40 (0.22, 0.72) *	0.45 (0.24, 0.84) *	< 0.01
	Model 2	0.96 (0.92, 1.01)	1.00	0.64 (0.33, 1.23)	0.36 (0.18, 0.76) *	0.42 (0.23, 0.79) *	0.02
	Model 3	0.96 (0.92, 1.00)	1.00	0.66 (0.34, 1.28)	0.34 (0.17, 0.70) *	0.38 (0.21, 0.71) *	< 0.01
	Model 4	0.94 (0.89, 0.99) *	1.00	1.10 (0.37, 3.31)	0.60 (0.21, 1.70)	0.48 (0.19, 1.18)	0.04

Note: Cd, cadmium; CI, confidence interval; Hg, mercury; OR, odds ratio; Pb, lead; Q1, Quartile 1 (as reference); Q2 - 4, Quartile 2 - 4; VE, vitamin E.

Model 1, adjusted for age, gender, and race; Model 2, adjusted for factors in Model 1 plus education, cotinine, body mass index, ratio of family income to poverty, and physical activity; Model 3, adjusted for factors in Model 2 plus hypertension and hypercholesteremia; Model 4, adjusted for factors in Model 3 plus medication histories of antidiabetic drugs and insulin. $N = 10{,}721$. * $P < 0.05$.

Table S3 Multiple-adjusted linear regressions of glucose with heavy metal exposure and VE intake in all adult participants.

		Continuous<br>β (95% CI)	Q1<br>(Reference)	Q2<br>β (95% CI)	Q3<br>β (95% CI)	Q4<br>β (95% CI)	P_{trend}
Ln concentrations of blood Pb (μg/dL), Cd, and Hg (μg/L)							
Ln-Pb	Model 1	-0.26 (-0.34, -0.19) *	1.00	-0.21 (-0.31, -0.10) *	-0.37 (-0.50, -0.24) *	-0.47 (-0.62, -0.33) *	< 0.01
	Model 2	-0.25 (-0.34, -0.16) *	1.00	-0.13 (-0.24, -0.01) *	-0.32 (-0.45, -0.18) *	-0.41 (-0.574, -0.24) *	< 0.01
	Model 3	-0.30 (-0.30, -0.20) *	1.00	-0.16 (-0.29, -0.03) *	-0.38 (-0.53, -0.24) *	-0.49 (-0.66, -0.32) *	< 0.01
	Model 4	-0.15 (-0.23, -0.07) *	1.00	-0.10 (-0.21, 0.01)	-0.19 (-0.31, -0.07) *	-0.23 (-0.39, -0.07) *	< 0.01
Ln-Cd	Model 1	-0.09 (-0.15, -0.04) *	1.00	-0.14 (-0.24, -0.03) *	-0.16 (-0.26, -0.05) *	-0.21 (-0.32, -0.10) *	< 0.01
	Model 2	-0.07 (-0.15, 0.01)	1.00	-0.07 (-0.20, 0.05)	-0.07 (-0.21, 0.06)	-0.11 (-0.27, 0.04)	0.16
	Model 3	-0.07 (-0.16, 0.02)	1.00	-0.06 (-0.20, 0.08)	-0.07 (-0.21, 0.08)	-0.11 (-0.29, 0.06)	0.202
	Model 4	-0.01 (-0.07, 0.06)	1.00	-0.01 (-0.12, 0.10)	0.01 (-0.11, 0.12)	0.02 (-0.11, 0.16)	0.757
Ln-Hg	Model 1	-0.07 (-0.11, -0.03) *	1.00	-0.10 (-0.21, 0.01) *	-0.17 (-0.28, -0.05) *	-0.19 (-0.30, -0.08) *	< 0.01
	Model 2	-0.03 (-0.07, 0.02)	1.00	-0.16 (-0.28, -0.05) *	-0.16 (-0.29, -0.03) *	-0.11 (-0.23, 0.01)	0.14
	Model 3	-0.04 (-0.09, 0.01)	1.00	-0.19 (-0.31, -0.06) *	-0.16 (-0.31, -0.01) *	-0.14 (-0.28, -0.01) *	0.11
	Model 4	0.01 (-0.03, 0.05)	1.00	-0.10 (-0.21, 0.01)	-0.09 (-0.21, 0.04)	-0.01 (-0.11, 0.10)	0.92
Intake, mg/day							
VE	Model 1	-0.01 (-0.02, -0.01) *	1.00	-0.23 (-0.46, -0.01) *	-0.24 (-0.47, -0.01) *	-0.30 (-0.54, -0.05) *	0.04
	Model 2	-0.010 (-0.01, -0.01) *	1.00	-0.14 (-0.35, 0.07)	-0.17 (-0.39, 0.06)	-0.25 (-0.45, -0.05) *	0.03
	Model 3	-0.01 (-0.02, -0.01) *	1.00	-0.16 (-0.39, 0.08)	-0.21 (-0.45, 0.03)	-0.23 (-0.52, -0.08) *	0.02
	Model 4	-0.01 (-0.01, -0.01) *	1.00	0.01 (-0.21, 0.23)	0.03 (-0.14, 0.20)	-0.09 (-0.25, 0.06)	0.14

Note: Cd, cadmium; CI, confidence interval; Hg, mercury; Pb, lead; Q1, Quartile 1 (as the reference group); Q2 - 4, Quartile 2 - 4; VE, vitamin E.

Model 1, adjusted for age, gender, and race; Model 2, adjusted for factors in Model 1 plus education, cotinine, body mass index, ratio of family income to poverty, and physical activity; Model 3, adjusted for factors in Model 2 plus hypertension and hypercholesteremia; Model 4, adjusted for factors in Model 3 plus medication histories of antidiabetic drugs and insulin. $N = 10{,}721$. * $P < 0.05$.

Table S4 Multiple-adjusted linear regressions of insulin with heavy metal exposures and VE intake in all adult participants.

		Continuous β (95% CI)	Q1 (Reference)	Q2 β (95% CI)	Q3 β (95% CI)	Q4 β (95% CI)	P_{trend}
Ln concentrations of blood Pb (μg/dL), Cd, and Hg (μg/L)							
Ln-Pb	Model 1	-1.97 (-2.77, -1.17) *	1.00	-1.84 (-2.91, -0.78) *	-2.83 (-4.02, -1.64) *	-3.45 (-5.15, -1.75) *	< 0.01
	Model 2	-0.82 (-1.47, -0.16) *	1.00	-1.07 (-2.06, -0.07) *	-1.60 (-2.68, -0.52) *	-1.15 (-2.35, 0.05)	0.03
	Model 3	-0.89 (-1.62, -0.16) *	1.00	-1.18 (-2.31, -0.04) *	-1.841 (-3.02, -0.66) *	-1.28 (-2.59, 0.04)	0.02
	Model 4	-0.64 (-1.26, -0.01) *	1.00	-1.08 (-2.18, 0.02)	-1.52 (-2.59, -0.45) *	-0.82 (-1.92, 0.29)	0.08
Ln-Cd	Model 1	-0.95 (-1.41, -0.48) *	1.00	-2.16 (-3.31, -1.02) *	-2.02 (-3.23, -0.82) *	-2.47 (-3.49, -1.44) *	< 0.01
	Model 2	-0.82 (-1.37, -0.27) *	1.00	-1.30 (-2.20, -0.39) *	-1.16 (-2.11, -0.21) *	-1.69 (-2.98, -0.40) *	0.01
	Model 3	-0.84 (-1.43, -0.25) *	1.00	-1.30 (-2.29, -0.32) *	-1.20 (-2.20, -0.19) *	-1.65 (-3.10, -0.20) *	0.02
	Model 4	-0.70 (-1.24, -0.16) *	1.00	-1.23 (-2.19, -0.26) *	-1.08 (-2.04, -0.13) *	-1.36 (-2.74, 0.02)	0.04
Ln-Hg	Model 1	-1.00 (-1.31, -0.68) *	1.00	-0.83 (-1.76, 0.09)	-1.33 (-2.26, -0.41) *	-2.61 (-3.57, -1.66) *	< 0.01
	Model 2	-0.20 (-0.45, 0.04)	1.00	-1.18 (-2.24, -0.12) *	-0.91 (-1.97, 0.14)	-0.88 (-1.77, 0.02)	0.01
	Model 3	-0.16 (-0.45, 0.12)	1.00	-1.18 (-2.41, 0.05)	-0.84 (-2.07, 0.40)	-0.93 (-1.97, 0.10)	0.14
	Model 4	-0.07 (-0.34, 0.21)	1.00	-0.96 (-2.09, 0.16)	-0.70 (-1.87, 0.48)	-0.63 (-1.54, 0.28)	0.29
Intake, mg/day							
VE	Model 1	-0.12 (-0.19, -0.05) *	1.00	-1.27 (-2.72, 0.19)	-1.35 (-3.11, 0.40)	-2.73 (-4.53, -0.93) *	< 0.01
	Model 2	-0.06 (-0.12, -0.01) *	1.00	-0.34 (-1.55, 0.87)	-0.04 (-1.51, 1.44)	-0.90 (-2.29, 0.50)	0.21
	Model 3	-0.07 (-0.13, -0.01) *	1.00	-0.66 (-1.98, 0.65)	-0.23 (-1.87, 1.41)	-1.35 (-2.80, 0.10)	0.08
	Model 4	-0.07 (-0.13, -0.01) *	1.00	-0.43 (-1.88, 1.01)	0.03 (-1.72, 1.77)	-1.11 (-2.65, 0.42)	0.14

Note: Cd, cadmium; CI, confidence interval; Hg, mercury; Pb, lead; Q1, Quartile 1 (as reference); Q2 - 4, Quartile 2 - 4; VE, vitamin E.

Model 1, adjusted for age, gender, and race; Model 2, adjusted for factors in Model 1 plus education, cotinine, body mass index, ratio of family income to poverty, and physical activity; Model 3, adjusted for factors in Model 2 plus hypertension and hypercholesteremia; Model 4, adjusted for factors in Model 3 plus medication histories of antidiabetic drugs and insulin. $N = 10{,}721$. * $P < 0.05$.

Table S5. Multiple-adjusted linear regressions of HOMA-IR with heavy metal exposures and VE intake in all adult participants.

		Continuous<br>*β* (95% CI)	Q1<br>(Reference)	Q2<br>*β* (95% CI)	Q3<br>*β* (95% CI)	Q4<br>*β* (95% CI)	P_{trend}
Ln concentrations of blood Pb (μg/dL), Cd, and Hg (μg/L)							
Ln-Pb	Model 1	-0.83 (-1.13, -0.53) *	1.00	-0.79 (-1.19, -0.39) *	-1.26 (-1.70, -0.83) *	-1.42 (-2.06, -0.77) *	< 0.01
	Model 2	-0.54 (-0.84, -0.24) *	1.00	-0.51 (-0.94, -0.09) *	-0.91 (-1.38, -0.43) *	-0.80 (-1.34, -0.26) *	< 0.01
	Model 3	-0.59 (-0.92, -0.26) *	1.00	-0.57 (-1.05, -0.09) *	-1.03 (-1.56, -0.49) *	-0.87 (-1.47, -0.27) *	< 0.01
	Model 4	-0.36 (-0.61, -0.10) *	1.00	-0.47 (-0.93, -0.02)	-0.73 (-1.16, -0.31) *	-0.46 (-0.92, 0.01) *	0.02
Ln-Cd	Model 1	-0.36 (-0.52, -0.19) *	1.00	-0.79 (-1.18, -0.40) *	-0.72 (-1.18, -0.25) *	-0.91 (-1.28, -0.55) *	< 0.01
	Model 2	-0.36 (-0.64, -0.08) *	1.00	-0.51 (-0.88, -0.13)	-0.49 (-0.90, -0.08) *	-0.71 (-1.33, -0.10) *	0.02
	Model 3	-0.37 (-0.68, -0.06) *	1.00	-0.51 (-0.92, -0.10)	-0.50 (-0.94, -0.06) *	-0.72 (-1.42, -0.03) *	0.03
	Model 4	-0.26 (-0.50, -0.01) *	1.00	-0.44 (-0.81, -0.07)	-0.39 (-0.76, -0.02)	-0.49 (-1.05, 0.08)	0.07
Ln-Hg	Model 1	-0.33 (-0.44, -0.21) *	1.00	-0.36 (-0.68, -0.04)	-0.48 (-0.79, -0.18) *	-0.87 (-1.24, -0.50) *	< 0.01
	Model 2	-0.07 (-0.16, 0.02)	1.00	-0.57 (-0.97, -0.17)	-0.39 (-0.79, 0.01)	-0.35 (-0.72, 0.02) *	0.13
	Model 3	-0.06 (-0.16, 0.04)	1.00	-0.60 (-1.07, -0.13)	-0.38 (-0.86, 0.10)	-0.40 (-0.82, 0.02) *	0.14
	Model 4	0.02 (-0.08, 0.12)	1.00	-0.44 (-0.85, -0.03)	-0.27 (-0.69, 0.16)	-0.15 (-0.49, 0.18)	0.65
Intake, mg/day							
VE	Model 1	-0.03 (-0.06, -0.01) *	1.00	-0.09 (-0.41, 0.23)	-0.20 (-0.55, 0.16)	-0.27 (-0.69, 0.14)	0.18
	Model 2	-0.02 (-0.04, -0.01) *	1.00	-0.04 (-0.38, 0.31)	-0.06 (-0.35, 0.23)	-0.13 (-0.51, 0.24)	0.50
	Model 3	-0.02 (-0.04, -0.01) *	1.00	-0.08 (-0.45, 0.30)	-0.06 (-0.38, 0.26)	-0.19 (-0.61, 0.24)	0.45
	Model 4	-0.02 (-0.036, -0.01) *	1.00	-0.01 (-0.40, 0.38)	-0.02 (-0.33, 0.29)	-0.15 (-0.53, 0.23)	0.44

Note: Cd, cadmium; CI, confidence interval; Hg, mercury; HOMA-IR, homeostatic model assessment for insulin resistance; Pb, lead; Q1, Quartile 1 (as reference); Q2 - 4, Quartile 2 - 4; VE, vitamin E.

Model 1, adjusted for age, gender, and race; Model 2, adjusted for factors in Model 1 plus education, cotinine, body mass index, ratio of family income to poverty, and physical activity; Model 3, adjusted for factors in Model 2 plus hypertension and hypercholesteremia; Model 4, adjusted for factors in Model 3 plus medication histories of antidiabetic drugs and insulin. N = 10,721. * $P < 0.05$.

Table S6. Multiple-adjusted linear regressions of HbA1c with heavy metal exposures and VE intake in all adult participants.

		Continuous<br>β (95% CI)	Q1<br>(Reference)	Q2<br>β (95% CI)	Q3<br>β (95% CI)	Q4<br>β (95% CI)	P_{trend}
Ln concentrations of blood Pb (μg/dL), Cd, and Hg (μg/L)							
Ln-Pb	Model 1	-0.13 (-0.16, -0.10) *	1.00	-0.10 (-0.16, -0.04) *	-0.17 (-0.24, -0.10) *	-0.25 (-0.32, -0.18) *	< 0.01
	Model 2	-0.13 (-0.17, -0.10) *	1.00	-0.07 (-0.13, -0.02) *	-0.16 (-0.22, -0.09) *	-0.23 (-0.31, -0.16) *	< 0.01
	Model 3	-0.15 (-0.19, -0.11) *	1.00	-0.09 (-0.16, -0.02) *	-0.18 (-0.25, -0.11) *	-0.26 (-0.34, -0.19) *	< 0.01
	Model 4	-0.06 (-0.10, -0.02) *	1.00	-0.05 (-0.11, 0.01)	-0.07 (-0.13, -0.01) *	-0.11 (-0.19, -0.04) *	< 0.01
Ln-Cd	Model 1	0.01 (-0.02, 0.04)	1.00	-0.05 (-0.11, 0.01)	-0.05 (-0.10, 0.01)	0.02 (-0.04, 0.08)	0.51
	Model 2	0.02 (-0.04, 0.05)	1.00	-0.03 (-0.09, 0.02)	-0.03 (-0.09, 0.03)	0.04 (-0.05, 0.12)	0.65
	Model 3	0.01 (-0.04, 0.05)	1.00	-0.02 (-0.08, 0.04)	-0.02 (-0.08, 0.04)	0.05 (-0.05, 0.14)	0.52
	Model 4	0.05 (0.01, 0.09) *	1.00	0.01 (-0.04, 0.05)	0.02 (-0.03, 0.07)	0.13 (0.05, 0.20) *	< 0.01
Ln-Hg	Model 1	-0.05 (-0.07, -0.04) *	1.00	-0.05 (-0.10, 0.01)	-0.09 (-0.15, -0.04) *	-0.14 (-0.19, -0.09) *	< 0.01
	Model 2	-0.02 (-0.04, -0.01) *	1.00	-0.06 (-0.12, -0.01) *	-0.07 (-0.14, -0.01) *	-0.07 (-0.14, -0.01) *	0.05
	Model 3	-0.03 (-0.05, -0.01) *	1.00	-0.07 (-0.14, -0.01) *	-0.08 (-0.15, -0.01) *	-0.08 (-0.15, -0.01) *	0.04
	Model 4	0.01 (-0.02, 0.02)	1.00	-0.02 (-0.08, 0.03)	-0.04 (-0.10, 0.02)	-0.01 (-0.06, 0.06)	0.89
Intake, mg/day							
VE	Model 1	-0.01 (-0.01, -0.01) *	1.00	-0.04 (-0.09, 0.02)	-0.06 (-0.12, 0.01)	-0.10 (-0.17, -0.03) *	< 0.01
	Model 2	-0.01 (-0.01, 0.01)	1.00	-0.01 (-0.07, 0.05)	0.01 (-0.06, 0.07)	-0.04 (-0.10, 0.03)	0.34
	Model 3	-0.01 (-0.01, 0.01)	1.00	-0.02 (-0.09, 0.05)	0.01 (-0.07, 0.07)	-0.05 (-0.12, 0.03)	0.29
	Model 4	-0.01 (-0.01, 0.01)	1.00	0.01 (-0.05, 0.05)	0.03 (-0.03, 0.09)	-0.03 (-0.09, 0.03)	0.38

Note: Cd, cadmium; CI, confidence interval; HbA1c, hemoglobin A1c; Hg, mercury; Pb, lead; Q1, Quartile 1 (as reference); Q2 - 4, Quartile 2 - 4; VE, vitamin E.
Model 1, adjusted for age, gender, and race; Model 2, adjusted for factors in Model 1 plus education, cotinine, body mass index, ratio of family income to poverty, and physical activity; Model 3, adjusted for factors in Model 2 plus hypertension and hypercholesteremia; Model 4, adjusted for factors in Model 3 plus medication histories of antidiabetic drugs and insulin. N = 10,721. * $P < 0.05$.

Table S7. Adjusted odds ratios of diabetes associated with heavy metal exposures and VE intake in participants aged > 65.

		Continuous OR (95% CI)	Q1 (Reference)	Q2 OR (95% CI)	Q3 OR (95% CI)	Q4 OR (95% CI)	P_{trend}
Ln concentrations of blood Pb (μg/dL), Cd, and Hg (μg/L)							
Ln-Pb	Model 1	0.70 (0.55, 0.89) *	1.00	0.65 (0.45, 0.95) *	0.55 (0.37, 0.80) *	0.53 (0.36, 0.78) *	< 0.01
	Model 2	0.61 (0.41, 0.88) *	1.00	0.44 (0.25, 0.76) *	0.50 (0.29, 0.85) *	0.48 (0.27, 0.85) *	0.02
	Model 3	0.63 (0.42, 0.93) *	1.00	0.41 (0.22, 0.73) *	0.49 (0.28, 0.87) *	0.52 (0.29, 0.93) *	0.05
Ln-Cd	Model 1	0.90 (0.72, 1.11)	1.00	0.75 (0.51, 1.10)	0.69 (0.47, 1.02)	0.91 (90.63, 1.31)	0.55
	Model 2	0.94 (0.67, 1.32)	1.00	0.77 90.44, 1.34)	0.92 (0.53, 1.58)	1.26 (0.71, 2.23)	0.43
	Model 3	0.94 (0.66, 1.33)	1.00	0.77 (0.43, 1.36)	0.90 (0.51, 1.57)	1.20 (0.66, 2.18)	0.56
Ln-Hg	Model 1	0.92 (0.80, 1.06)	1.00	1.12 (0.77, 1.62)	0.88 (0.60, 1.31)	0.88 (0.59, 1.29)	0.31
	Model 2	0.93 90.75, 1.15)	1.00	1.07 (0.61, 1.89)	0.84 (0.46, 1.55)	1.04 (0.59, 1.88)	0.94
	Model 3	0.94 (0.75, 1.17)	1.00	1.05 90.58, 1.91)	0.79 (0.42, 1.50)	1.07 (0.58, 1.98)	0.99
Intake, mg/day							
VE	Model 1	0.99 (0.96, 1.01)	1.00	0.75 (0.51, 1.07)	0.51 (90.34, 0.76) *	0.73 (0.50, 1.05)	0.03
	Model 2	0.97 (0.93, 1.01)	1.00	0.59 (0.34, 1.02)	0.40 (90.22, 0.73) *	0.54 (0.31, 0.95) *	0.02
	Model 3	0.97 (0.93, 1.01)	1.00	0.59 (0.33, 1.05)	0.44 (0.23, 0.81) *	0.55 (0.31, 0.99) *	0.04

Note: Cd, cadmium; CI, confidence interval; Hg, mercury; OR, odds ratio; Pb, lead; Q1, Quartile 1 (as reference); Q2 - 4, Quartile 2 - 4; VE, vitamin E.

Model 1, adjusted for age, gender, and race;

Model 2, adjusted for factors in Model 1 plus education, cotinine, body mass index, ratio of family income to poverty, and physical activity;

Model 3, adjusted for factors in Model 2 plus hypertension and hypercholesteremia.

$N = 1{,}833$.

* $P < 0.05$.

Table S8. Multiple-adjusted linear regressions of glucose with heavy metal exposures and VE intake in participants aged > 65.

		Continuous<br>β (95% CI)	Q1<br>(Reference)	Q2<br>β (95% CI)	Q3<br>β (95% CI)	Q4<br>β (95% CI)	P_{trend}
Ln concentrations of blood Pb (μg/dL), Cd, and Hg (μg/L)							
Ln-Pb	Model 1	-0.12 (-0.21, -0.03) *	1.00	-0.09 (-0.23, 0.05)	-0.13 (-0.28, 0.00) *	-0.17 (-0.31, -0.02) *	0.01
	Model 2	-0.15 (-0.27, -0.04) *	1.00	-0.23 (-0.41, -0.05) *	-0.20 (-0.38, -0.02) *	-0.20 (-0.38, -0.01) *	0.05
	Model 3	-0.16 (-0.26, -0.05) *	1.00	-0.26 (-0.43, -0.09) *	-0.25 (-0.41, -0.08) *	-0.20 (-0.38, -0.03) *	0.03
Ln-Cd	Model 1	-0.02 (-0.10, 0.06)	1.00	-0.04 (-0.18, 0.10)	0.02 (-0.12, 0.16)	-0.00 (-0.14, 0.13)	0.83
	Model 2	-0.01 (-0.12, 0.11)	1.00	0.01 (-0.16, 0.18)	0.00 (-0.17, 0.17)	0.08 (-0.10, 0.28)	0.47
	Model 3	-0.02 (-0.12, 0.08)	1.00	-0.02 (-0.18, 0.13)	-0.00 (-0.16, 0.16)	0.05 (-0.12, 0.23)	0.57
Ln-Hg	Model 1	-0.01 (-0.06, 0.05)	1.00	0.03 (-0.11, 0.17)	-0.03 (-0.18, 0.10)	-0.00 (-0.15, 0.13)	0.68
	Model 2	0.01 (-0.06, 0.08)	1.00	-0.01 (-0.20, 0.17)	-0.06 (-0.25, 0.12)	0.01 (-0.17, 0.20)	0.97
	Model 3	0.01 (-0.06, 0.07)	1.00	-0.04 (-0.22, 0.12)	-0.13 (-0.31, 0.04)	-0.00 (-0.18, 0.17)	0.84
Intake, mg/day							
VE	Model 1	-0.01 (-0.02, 0.01)	1.00	0.01 (-0.13, 0.15)	-0.18 (-0.32, -0.04) *	-0.10 (-0.24, 0.03)	0.03
	Model 2	-0.01 (-0.02, 0.01)	1.00	-0.13 (-0.32, 0.05)	-0.26 (-0.45, -0.07) *	-0.14 (-0.33, 0.04)	0.11
	Model 3	-0.01 (-0.01, 0.01)	1.00	-0.04 (-0.21, 0.13)	-0.18 (-0.36, 0.00)	-0.05 (-0.23, 0.11)	0.35

Note: Cd, cadmium; CI, confidence interval; Hg, mercury; Pb, lead; Q1, Quartile 1 (as reference); Q2 - 4, Quartile 2 - 4; VE, vitamin E.

Model 1, adjusted for age, gender, and race;

Model 2, adjusted for factors in Model 1 plus education, cotinine, body mass index, ratio of family income to poverty, and physical activity;

Model 3, adjusted for factors in Model 2 plus hypertension and hypercholesteremia.

$N = 1,833$.

* $P < 0.05$.

Table S9. Basic profiles of participants aged 18 - 65 by diabetes, NHANES 2007 - 2018.

	**All**	**No-diabetes**	**Diabetes**	***P****
N	7,601	7,214	387	
**Age, y, means ± SD**	40.58 ± 14.12	40.06 ± 14.06	50.42 ± 11.21	< 0.01
**Gender, *n* (%)**				< 0.01
male	3,692 (48.6)	3,453 (47.9)	239 (61.8)	
female	3,909 (51.4)	3,761 (52.1)	148 (38.2)	
**Body mass index (BMI), *n* (%)**				< 0.01
BMI < 25 kg/m^2	2,423 (32.2)	2,373 (33.2)	50 (13.0)	
25 ≤ BMI < 30 kg/m^2	2,465 (32.7)	2,364 (33.1)	101 (26.3)	
BMI ≥ 30 kg/m^2	2,646 (35.1)	2,413 (33.7)	233 (60.7)	
**Race, *n* (%)**				< 0.01
Mexican american	1,263 (16.6)	1,180 (16.4)	83 (21.4)	
other Hispanic	855 (11.2)	793 (11.0)	62 (16.0)	
non-Hispanic white	2,989 (39.3)	2,875 (39.9)	114 (29.5)	
non-Hispanic black	1,617 (21.3)	1,522 (21.1)	95 (24.5)	
other races	877 (11.5)	844 (11.7)	33 (8.5)	
**Education, *n* (%)**				< 0.01
< 9th grade	534 (7.0)	479 (6.6)	55 (14.2)	
9-11th grade	942 (12.4)	880 (12.2)	62 (16.0)	
high school graduate	1,757 (23.1)	1,654 (22.9)	103 (26.6)	
college level graduate	2,350 (30.9)	2,250 (31.2)	100 (25.8)	
≥ college graduate	2,013 (26.5)	1,946 (27.0)	67 (17.3)	
**RFIP**	2.48 (1.65)	2.48 (1.65)	2.43 (1.65)	0.54
**Physical activity, *n* (%)**				< 0.01
high	2,917 (47.6)	2,824 (48.2)	93 (35.4)	
middle	3,191 (52.1)	3,023 (51.6)	168 (63.9)	
low	19 (0.3)	17 (0.3)	2 (0.8)	
**Circulating parameters, means ± SD**				
serum cotinine, ng/mL	59.14 ± 126.61	59.15 ± 126.48	58.96 ± 129.09	0.97
blood Pb, μg/dL	1.36 ± 1.54	1.35 ± 1.54	1.56 ± 1.39	0.01
blood Cd, μg/L	0.50 ± 0.61	0.50 ± 0.61	0.50 ± 0.61	0.92
blood Hg, μg/L	1.46 ± 2.21	1.45 ± 2.18	1.66 ± 2.76	0.06
plasma glucose, mmol/L	5.58 ± 1.11	5.43 ± 0.54	8.40 ± 3.20	< 0.01
serum insulin, μU/mL	13.11 ± 11.78	12.51 ± 10.61	24.26 ± 22.21	< 0.01
HOMA-IR	3.39 ± 3.63	3.09 ± 2.80	8.83 ± 8.99	< 0.01
blood HbA1c, %	5.46 ± 0.65	5.39 ± 0.38	6.90 ± 1.87	< 0.01
**Nutrient intakes, means ± SD**				
VA, μg/day	596.84 ± 515.19	599.27 ± 521.89	551.52 ± 366.13	0.07
VC, mg/day	85.18 ± 81.75	85.24 ± 82.00	84.01 ± 77.06	0.77
VE, mg/day	8.66 ± 6.51	8.67 ± 6.53	8.37 ± 5.96	0.37
selenium, μg/day	115.28 ± 54.34	115.07 ± 54.13	119.24 ± 57.97	0.14

Note: Cd, cadmium; HbA1c, hemoglobin A1c; Hg, mercury; HOMA-IR, homeostatic model assessment for insulin resistance; Pb, lead; RFIP, the ratio of family income to poverty; SD, standard deviations; VA,

vitamin A; VC, vitamin C; VE, vitamin E. * Between diabetes and non-diabetes.

Table S10. Multiple-adjusted linear regressions of HbA1c with heavy metal exposures and VE intake in participants aged 18 - 65.

		Continuous<br>β (95% CI)	Q1<br>(Reference)	Q2<br>β (95% CI)	Q3<br>β (95% CI)	Q4<br>β (95% CI)	P_{trend}
Ln concentrations of blood Pb (μg/dL), Cd, and Hg (μg/L)							
Ln-Pb	Model 1	0.11 (0.09, 0.13) *	1.00	0.06 (0.02, 0.09) *	0.12 (0.08, 0.15) *	0.22 (0.18, 0.26) *	< 0.01
	Model 2	0.10 (0.07, 0.12) *	1.00	0.08 (0.04, 0.11) *	0.12 (0.08, 0.16) *	0.23 (0.18, 0.27) *	< 0.01
	Model 3	0.08 (0.06, 0.11) *	1.00	0.08 (0.04, 0.12) *	0.10 (0.06, 0.15) *	0.20 (0.15, 0.25) *	< 0.01
Ln-Cd	Model 1	0.09 (0.07, 0.11) *	1.00	0.05 (0.01, 0.09) *	0.10 (0.07, 0.14) *	0.19 (0.15, 0.23) *	< 0.01
	Model 2	0.09 (0.06, 0.13) *	1.00	0.06 (0.01, 0.10) *	0.10 (0.06, 0.14) *	0.19 (0.12, 0.26) *	< 0.01
	Model 3	0.09 (0.05, 0.13) *	1.00	0.06 (0.02, 0.11) *	0.10 (0.06, 0.14) *	0.20 (0.12, 0.27) *	< 0.01
Ln-Hg	Model 1	0.01 (-0.01, 0.03)	1.00	0.03 (-0.01, 0.07)	0.03 (-0.01, 0.07)	0.02 (-0.03, 0.06)	0.51
	Model 2	0.03 (0.01, 0.05) *	1.00	0.02 (-0.02, 0.07)	0.03 (-0.02, 0.08)	0.06 (0.01, 0.12) *	0.15
	Model 3	0.02 (0.01, 0.04) *	1.00	0.02 (-0.03, 0.08)	0.02 (-0.04, 0.08)	0.04 (-0.01, 0.10)	0.17
Intake, mg/day							
VE	Model 1	-0.01 (-0.01, -0.01) *	1.00	-0.01 (-0.08, 0.05)	0.08 (-0.01, 0.15)	-0.02 (-0.09, 0.05)	0.89
	Model 2	-0.01 (-0.01, 0.01)	1.00	0.05 (-0.02, 0.11)	0.12 (0.04, 0.20)	0.07 (-0.01, 0.14)	0.10
	Model 3	-0.01 (-0.01, 0.01)	1.00	0.04 (-0.03, 0.11)	0.09 (0.01, 0.17)	0.02 (-0.05, 0.10)	0.73

Note: Cd, cadmium; CI, confidence interval; HbA1c, hemoglobin A1c; Hg, mercury; Pb, lead; Q1, Quartile 1 (as reference); Q2 - 4, Quartile 2 - 4; VE, vitamin E.

Model 1, adjusted for age, gender, and race;

Model 2, adjusted for factors in Model 1 plus education, cotinine, body mass index, ratio of family income to poverty, and physical activity;

Model 3, adjusted for factors in Model 2 plus hypertension and hypercholesteremia.

$N = 7{,}601$.

* $P < 0.05$.

Table S11. Multiple-adjusted linear regressions of insulin with heavy metal exposures and VE intake in participants aged 18 - 65.

		Continuous β (95% CI)	Q1 (Reference)	Q2 β (95% CI)	Q3 β (95% CI)	Q4 β (95% CI)	P_{trend}
Ln concentrations of blood Pb (μg/dL), Cd, and Hg (μg/L)							
Ln-Pb	Model 1	-0.94 (-1.54, -0.34) *	1.00	-1.43 (-2.39, -0.46) *	-2.00 (-3.10, -0.90) *	-2.23 (-3.41, -1.04) *	< 0.01
	Model 2	-0.47 (-0.93, -0.01) *	1.00	-1.29 (-2.21, -0.36) *	-1.84 (-2.85, -0.82) *	-1.15 (-2.18, -0.13) *	0.02
	Model 3	-0.59 (-1.10, -0.08) *	1.00	-1.40 (-2.48, -0.33) *	-2.24 (-3.37, -1.12) *	-1.31 (-2.45, -0.17) *	0.01
Ln-Cd	Model 1	-0.65 (-1.11, -0.20) *	1.00	-0.77 (-1.98, 0.44)	-1.32 (-2.37, -0.28) *	-1.64 (-2.65, -0.64) *	0.01
	Model 2	-0.62 (-1.12, -0.11) *	1.00	-0.70 (-1.55, 0.14)	-0.82 (-1.79, 0.16)	-1.33 (-2.28, -0.39) *	0.01
	Model 3	-0.66 (-1.21, -0.12) *	1.00	-0.66 (-1.62, 0.31)	-0.85 (-1.93, 0.23)	-1.32 (-2.34, -0.29) *	0.02
Ln-Hg	Model 1	-0.93 (-1.23, -0.62) *	1.00	-0.56 (-1.52, 0.41)	-1.08 (-2.02, -0.14) *	-2.27 (-3.09, -1.45) *	< 0.01
	Model 2	-0.20 (-0.42, 0.03)	1.00	-0.75 (-1.54, 0.05)	-0.62 (-1.37, 0.14)	-0.50 (-1.13, 0.14)	0.20
	Model 3	-0.16 (-0.44, 0.12)	1.00	-0.66 (-1.56, 0.23)	-0.47 (-1.30, 0.36)	-0.49 (-1.24, 0.26)	0.31
Intake, mg/day							
VE	Model 1	-0.15 (-0.23, -0.08) *	1.00	-0.97 (-2.64, 0.70)	-1.02 (-2.86, 0.82)	-3.11 (-4.98, -1.24) *	< 0.01
	Model 2	-0.09 (-0.15, -0.03) *	1.00	-0.09 (-1.36, 1.19)	0.05 (-1.45, 1.55)	-1.16 (-2.61, 0.29)	0.09
	Model 3	-0.096 (-0.16, -0.03) *	1.00	-0.39 (-1.89, 1.11)	-0.09 (-1.83, 1.66)	-1.63 (-3.27, 0.02)	0.04

Note: Cd, cadmium; CI, confidence interval; Hg, mercury; Pb, lead; Q1, Quartile 1 (as reference); Q2 - 4, Quartile 2 - 4; VE, vitamin E.

Model 1, adjusted for age, gender, and race;

Model 2, adjusted for factors in Model 1 plus education, cotinine, body mass index, ratio of family income to poverty, and physical activity;

Model 3, adjusted for factors in Model 2 plus hypertension and hypercholesteremia.

$N = 7,601$.

* $P < 0.05$.

Table S12. Multiple-adjusted linear regressions of HOMA-IR with heavy metal exposures and VE intake in participants aged 18 - 65.

		Continuous β (95% CI)	Q1 (Reference)	Q2 β (95% CI)	Q3 β (95% CI)	Q4 β (95% CI)	P_{trend}
Ln concentrations of blood Pb (μg/dL), Cd, and Hg (μg/L)							
Ln-Pb	Model 1	-0.20 (-0.37, -0.03) *	1.00	-0.38 (-0.64, -0.11) *	-0.47 (-0.78, -0.17) *	-0.45 (-0.78, -0.13) *	< 0.01
	Model 2	-0.09 (-0.22, 0.05)	1.00	-0.30 (-0.56, -0.05) *	-0.42 (-0.70, -0.14) *	-0.16 (-0.44, 0.12)	0.20
	Model 3	-0.13 (-0.28, 0.02)	1.00	-0.32 (-0.62, -0.03) *	-0.55 (-0.85, -0.24) *	-0.21 (-0.52, 0.11)	0.10
Ln-Cd	Model 1	-0.15 (-0.28, -0.03) *	1.00	-0.17 (-0.53, 0.19)	-0.32 (-0.62, -0.02) *	-0.38 (-0.66, -0.11) *	< 0.01
	Model 2	-0.12 (-0.27, 0.02)	1.00	-0.16 (-0.40, 0.07)	-0.17 (-0.44, 0.10)	-0.27 (-0.52, -0.02) *	0.06
	Model 3	-0.14 (-0.29, 0.01)	1.00	-0.15 (-0.41, 0.11)	-0.19 (-0.48, 0.11)	-0.27 (-0.55, -0.00) *	0.06
Ln-Hg	Model 1	-0.22 (-0.30, -0.14) *	1.00	-0.14 (-0.41, 0.13)	-0.27 (-0.51, -0.02) *	-0.54 (-0.77, -0.31) *	< 0.01
	Model 2	-0.02 (-0.08, 0.04)	1.00	-0.23 (-0.45, -0.01) *	-0.16 (-0.36, 0.05)	-0.07 (-0.25, 0.11)	0.64
	Model 3	-0.01 (-0.09, 0.06)	1.00	-0.20 (-0.45, 0.05)	-0.11 (-0.33, 0.12)	-0.08 (-0.29, 0.13)	0.68
Intake, mg/day							
VE	Model 1	-0.04 (-0.06, -0.02) *	1.00	-0.24 (-0.69, 0.21)	-0.24 (-0.72, 0.25)	-0.80 (-1.30, -0.30) *	< 0.01
	Model 2	-0.02 (-0.04, -0.01) *	1.00	-0.01 (-0.33, 0.32)	0.050 (-0.34, 0.44)	-0.26 (-0.64, 0.12)	0.14
	Model 3	-0.03 (-0.04, -0.01) *	1.00	-0.09 (-0.47, 0.28)	-0.01 (-0.45, 0.44)	-0.41 (-0.84, 0.02)	0.04

Note: Cd, cadmium; CI, confidence interval; Hg, mercury; HOMA-IR, homeostatic model assessment for insulin resistance; Pb, lead; Q1, Quartile 1 (as reference); Q2 - 4, Quartile 2 - 4; VE, vitamin E.

Model 1, adjusted for age, gender, and race;

Model 2, adjusted for factors in Model 1 plus education, cotinine, body mass index, ratio of family income to poverty, and physical activity;

Model 3, adjusted for factors in Model 2 plus hypertension and hypercholesteremia.

N = 7,601.

* $P < 0.05$.

Table S13. Logistic regression between heavy metals and diabetes in men aged 18 - 65.

		Continuous OR (95% CI)	Q1 (Reference)	Q2 OR (95% CI)	Q3 OR (95% CI)	Q4 OR (95% CI)	P_{trend}
Ln concentrations of blood Pb (μg/dL), Cd, and Hg (μg/L)							
Ln-Pb	Model 1	1.29 (1.03, 1.61) *	1.00	1.16 (0.66, 2.04)	1.69 (0.99, 2.90)	1.89 (1.12, 3.19) *	< 0.01 *
	Model 2	1.33 (1.02, 1.75) *	1.00	1.21 (0.63, 2.34)	1.76 (0.90, 3.46)	2.18 (1.14, 4.19) *	0.01 *
	Model 3	1.20 (0.86, 1.69)	1.00	1.14 (0.56, 2.30)	1.37 (0.65, 2.89)	1.82 (0.85, 3.91)	0.10
Ln-Cd	Model 1	1.18 (0.95, 1.46)	1.00	1.11 (0.60, 2.06)	1.59 (0.92, 2.75)	1.47 (0.79, 2.73)	0.09
	Model 2	1.36 (0.972, 1.891)	1.00	1.31 (0.62, 2.78)	1.87 (0.91, 3.83)	2.16 (0.84, 5.58)	0.04 *
	Model 3	1.27 (0.88, 1.82)	1.00	1.37 (0.63, 2.97)	1.95 (0.92, 4.13)	1.81 (0.66, 4.96)	0.12
Ln-Hg	Model 1	1.19 (1.00, 1.41)	1.00	0.88 (0.53, 1.47)	1.58 (0.95, 2.62)	1.41 (0.82, 2.41)	0.06
	Model 2	1.24 (0.98, 1.57)	1.00	0.70 (0.37, 1.33)	1.64 (0.87, 3.09)	1.43 (0.705, 2.91)	0.10
	Model 3	1.20 (0.93, 1.56)	1.00	0.67 (0.33, 1.38)	1.68 (0.80, 3.52)	1.29 (0.58, 2.87)	0.19
Intake, mg/day							
VE	Model 1	0.93 (0.87, 1.00) *	1.00	2.00 (0.63, 6.32)	1.22 (0.44, 3.39)	0.53 (0.24, 1.19)	0.04
	Model 2	0.93 (0.85, 1.02)	1.00	2.63 (0.72, 9.64)	1.69 (0.50, 5.69)	0.59 (0.19, 1.86)	0.11
	Model 3	0.93 (0.85, 1.01)	1.00	3.02 (0.73, 12.61)	1.75 (0.48, 6.38)	0.61 (0.18, 2.13)	0.06

Note: Cd, cadmium; CI, confidence interval; Hg, mercury; OR, odds ratio; Pb, lead; Q1, Quartile 1 (as reference); Q2 - 4, Quartile 2 - 4; VE, vitamin E.

Model 1, adjusted for age, gender, and race;

Model 2, adjusted for factors in Model 1 plus education, cotinine, body mass index, ratio of family income to poverty, and physical activity;

Model 3, adjusted for factors in Model 2 plus hypertension and hypercholesteremia.

$N = 3,692$.

* $P < 0.05$.

Table S14. Logistic regression between heavy metals and diabetes in women aged 18 - 65.

		Continuous<br>OR (95% CI)	Q1<br>(Reference)	Q2<br>OR (95% CI)	Q3<br>OR (95% CI)	Q4<br>OR (95% CI)	P_{trend}
Ln concentrations of blood Pb (μg/dL), Cd, and Hg (μg/L)							
Ln-Pb	Model 1	1.14 (0.79, 1.62)	1.00	0.96 (0.98, 3.91)	1.28 (0.69, 2.39)	1.88 (1.08, 3.27) *	0.13
	Model 2	1.26 (0.70, 2.26)	1.00	2.75 (1.00, 7.60)	1.89 (0.72, 4.95)	2.56 (0.96, 6.83)	0.15
	Model 3	1.13 (0.58, 2.18)	1.00	2.77 (1.02, 7.53) *	1.42 (0.46, 4.38)	2.27 (0.80, 6.45)	0.42
Ln-Cd	Model 1	1.10 (0.87, 1.38)	1.00	1.11 (0.58, 2.12)	1.31 (0.64, 2.69)	1.22 (0.72, 2.08)	0.36
	Model 2	1.38 (0.86, 2.20)	1.00	1.46 (0.58, 3.68)	1.79 (0.72, 4.41)	1.86 (0.75, 4.62)	0.12
	Model 3	1.35 (0.84, 2.19)	1.00	1.42 (0.54, 3.73)	1.54 (0.61, 3.85)	1.89 (0.76, 4.69)	0.18
Ln-Hg	Model 1	0.99 (0.80, 1.22)	1.00	1.23 (0.62, 2.45)	1.00 (0.49, 2.03)	1.03 (0.52, 2.05)	0.90
	Model 2	1.05 (0.79, 1.39)	1.00	1.49 (0.58, 3.81)	1.06 (0.38, 2.91)	1.11 (0.41, 3.05)	0.97
	Model 3	0.98 (0.73, 1.32)	1.00	1.62 (0.64, 4.10)	1.04 (0.36, 3.00)	0.83 (0.28, 2.48)	0.56
Intake, mg/day							
VE	Model 1	0.95 (0.88, 1.02)	1.00	0.82 (0.19, 3.55)	0.97 (0.20, 4.62)	0.41 (0.10, 1.74)	0.31
	Model 2	0.88 (0.76, 1.01)	1.00	0.65 (0.13, 3.39)	0.47 (0.06, 3.45)	0.20 (0.03, 1.27)	0.11
	Model 3	0.82 (0.72, 0.94) *	1.00	0.65 (0.12, 3.57)	0.42 (0.06, 3.06)	0.12 (0.02, 0.74) *	0.03

Note: Cd, cadmium; CI, confidence interval; Hg, mercury; OR, odds ratio; Pb, lead; Q1, Quartile 1 (as reference); Q2 - 4, Quartile 2 - 4; VE, vitamin E.

Model 1, adjusted for age, gender, and race;

Model 2, adjusted for factors in Model 1 plus education, cotinine, body mass index, ratio of family income to poverty, and physical activity;

Model 3, adjusted for factors in Model 2 plus hypertension and hypercholesteremia.

$N = 3,909$.

* $P < 0.05$.

Table S15. Logistic regression between heavy metals and risk of diabetes in participants aged 18 - 65 with BMI < 25 kg/m^2.

		Continuous OR (95% CI)	Q1 (Reference)	Q2 OR (95% CI)	Q3 OR (95% CI)	Q4 OR (95% CI)	P_{trend}
Ln concentrations of blood Pb (μg/dL), Cd, and Hg (μg/L)							
Ln-Pb	Model 1	1.40 (0.94, 2.05) *	1.00	1.26 (0.41, 4.27)	2.39 (0.91, 7.54)	2.99 (1.17, 9.28) *	< 0.01 *
	Model 2	1.26 (0.69, 2.29)	1.00	1.07 (0.23, 6.05)	1.51 (0.39, 8.08)	2.47 (0.68, 12.80)	0.10
	Model 3	1.19 (0.61, 2.30)	1.00	0.70 (0.13, 4.19)	0.88 (0.20, 4.96)	1.73 (0.46, 9.18)	0.19
Ln-Cd	Model 1	1.24 (0.92, 1.67)	1.00	2.11 (0.87, 5.54)	2.86 (1.22, 7.42)	2.24 (0.95, 5.82)	0.08
	Model 2	1.15 (0.72, 1.87)	1.00	1.70 (0.53, 5.92)	2.30 (0.77, 7.80)	1.37 (0.40, 5.28)	0.52
	Model 3	1.04 (0.60, 1.82)	1.00	2.34 (0.67, 9.34)	2.32 (0.68, 9.33)	1.07 (0.25, 5.29)	0.81
Ln-Hg	Model 1	1.14 (0.87, 1.48)	1.00	1.85 (0.83, 4.39)	1.47 (0.63, 3.60)	1.42 (0.59, 3.56)	0.61
	Model 2	1.18 (0.83, 1.69)	1.00	2.29 (0.81, 7.50)	1.92 (0.64, 6.51)	1.96 (0.62, 6.92)	0.37
	Model 3	1.15 (0.78, 1.69)	1.00	2.80 (0.86, 11.07)	2.24 (0.66, 9.07)	1.61 (0.43, 6.95)	0.67
Intake, mg/day							
VE	Model 1	0.96 (0.90, 1.01)	1.00	1.03 (0.47, 2.27)	1.04 (0.48, 2.31)	0.50 (0.19, 1.24)	0.17
	Model 2	0.95 (0.88, 1.02)	1.00	0.88 (0.31, 2.49)	1.00 (0.36, 2.82)	0.44 (0.12, 1.47)	0.25
	Model 3	0.92 (0.82, 0.99) *	1.00	0.75 (0.22, 2.49)	0.77 (0.22, 2.60)	0.26 (0.05, 1.06)	0.06

Note: BMI, body mass index; Cd, cadmium; CI, confidence interval; Hg, mercury; OR, odds ratio; Pb, lead; Q1, Quartile 1 (as reference); Q2 - 4, Quartile 2 - 4; VE, vitamin E.

Model 1, adjusted for age, gender, and race;

Model 2, adjusted for factors in Model 1 plus education, cotinine, body mass index, ratio of family income to poverty, and physical activity;

Model 3, adjusted for factors in Model 2 plus hypertension and hypercholesteremia.

$N = 2{,}450$.

* $P < 0.05$.

Table S16. Logistic regression between heavy metals and risk of diabetes in participants aged 18 - 65 with BMI ≥ 25 kg/m^2.

		Continuous OR (95% CI)	Q1 (Reference)	Q2 OR (95% CI)	Q3 OR (95% CI)	Q4 OR (95% CI)	P_{trend}
Ln concentrations of blood Pb (μg/dL), Cd, and Hg (μg/L)							
Ln-Pb	Model 1	1.14 (0.93, 1.41)	1.00	1.23 (0.77, 1.96)	1.30 (0.86, 1.96)	1.77 (1.16, 2.68) *	0.01
	Model 2	1.09 (0.82, 1.46)	1.00	1.65 (0.88, 3.10)	1.51 (0.82, 2.79)	2.07 (1.14, 3.75) *	0.04
	Model 3	0.98 (0.68, 1.41)	1.00	1.50 (0.77, 2.91)	1.22 (0.61, 2.47)	1.64 (0.82, 3.26)	0.36
Ln-Cd	Model 1	1.12 (0.94, 1.33)	1.00	1.18 (0.73, 1.90)	1.25 (0.78, 2.00)	1.26 (0.83, 1.92)	0.26
	Model 2	1.17 (0.85, 1.62)	1.00	1.29 (0.69, 2.38)	1.20 (0.66, 2.17)	1.40 (0.72, 2.74)	0.37
	Model 3	1.10 (0.78, 1.54)	1.00	1.42 (0.73, 2.76)	1.23 (0.65, 2.35)	1.25 (0.61, 2.56)	0.54
Ln-Hg	Model 1	1.12 (0.96, 1.31)	1.00	0.90 (0.58, 1.38)	1.03 (0.64, 1.66)	1.48 (0.96, 2.27)	0.08
	Model 2	1.12 (0.91, 1.38)	1.00	0.87 (0.50, 1.54)	1.03 (0.53, 2.00)	1.44 (0.78, 2.65)	0.22
	Model 3	1.08 (0.86, 1.36)	1.00	0.87 (0.46, 1.62)	1.04 (0.51, 2.15)	1.30 (0.64, 2.64)	0.38
Intake, mg/day							
VE	Model 1	0.95 (0.88, 1.02)	1.00	0.99 (0.32, 3.02)	0.46 (0.18, 1.16)	0.50 (0.21, 1.20)	0.10
	Model 2	0.93 (0.83, 1.03)	1.00	1.12 (0.32, 3.83)	0.30 (0.09, 1.01)	0.41 (0.13, 1.30)	0.08
	Model 3	0.93 (0.83, 1.03)	1.00	1.08 (0.29, 4.04)	0.27 (0.08, 0.94) *	0.39 (0.13, 1.22)	0.05

Note: BMI, body mass index; Cd, cadmium; CI, confidence interval; Hg, mercury; OR, odds ratio; Pb, lead; Q1, Quartile 1 (as reference); Q2 - 4, Quartile 2 - 4; VE, vitamin E.

Model 1, adjusted for age, gender, and race;

Model 2, adjusted for factors in Model 1 plus education, cotinine, body mass index, ratio of family income to poverty, and physical activity;

Model 3, adjusted for factors in Model 2 plus hypertension and hypercholesteremia.

$N = 5,084$.

* $P < 0.05$.

Table S17. Multiple-adjusted linear regression between heavy metals and glucose in men aged 18 - 65.

		Continuous<br>β (95% CI)	Q1<br>(Reference)	Q2<br>β (95% CI)	Q3<br>β (95% CI)	Q4<br>β (95% CI)	P trend
Ln concentrations of blood Pb (μg/dL), Cd, and Hg (μg/L)							
Ln-Pb	Model 1	0.08 (0.02, 0.14) *	1.00	0.05 (-0.04, 0.13)	0.12 (0.03, 0.21) *	0.20 (0.10, 0.30) *	< 0.01
	Model 2	0.08 (0.02, 0.14) *	1.00	0.04 (-0.05, 0.13)	0.12 (0.02, 0.21) *	0.21 (0.09, 0.34) *	< 0.01
	Model 3	0.04 (-0.02, 0.11)	1.00	0.01 (-0.09, 0.11)	0.05 (-0.05, 0.15)	0.17 (0.02, 0.32) *	0.02
Ln-Cd	Model 1	0.02 (-0.03, 0.06)	1.00	0.04 (-0.08, 0.15)	0.09 (-0.02, 0.19)	0.06 (-0.06, 0.17)	0.21
	Model 2	0.06 (-0.01, 0.13)	1.00	0.11 (-0.02, 0.24)	0.14 (0.02, 0.25) *	0.19 (0.01, 0.349) *	0.03
	Model 3	0.07 (-0.01, 0.14)	1.00	0.12 (-0.03, 0.26)	0.15 (0.03, 0.27) *	0.18 (0.01, 0.36) *	0.02
Ln-Hg	Model 1	0.02 (-0.01, 0.06)	1.00	0.01 (-0.08, 0.11)	0.04 (-0.07, 0.15)	0.03 (-0.07, 0.12)	0.48
	Model 2	0.04 (0.01, 0.08) *	1.00	0.01 (-0.11, 0.11)	0.04 (-0.09, 0.16)	0.06 (-0.05, 0.17)	0.23
	Model 3	0.03 (-0.01, 0.08)	1.00	-0.02 (-0.15, 0.11)	0.05 (-0.10, 0.21)	0.03 (-0.10, 0.16)	0.47
Intake, mg/day							
VE	Model 1	-0.01 (-0.01, 0.01)	1.00	0.19 (0.01, 0.39) *	0.08 (-0.11, 0.26)	0.01 (-0.15, 0.17)	0.31
	Model 2	-0.002 (-0.010, 0.006)	1.00	0.23 (0.04, 0.42) *	0.16 (-0.02, 0.34)	0.09 (-0.8, 0.26)	0.97
	Model 3	-0.003 (-0.012, 0.006)	1.00	0.19 (-0.03, 0.41)	0.10 (-0.10, 0.30)	0.05 (-0.15, 0.25)	0.67

Note: Cd, cadmium; CI, confidence interval; Hg, mercury; Pb, lead; Q1, Quartile 1 (as reference); Q2 - 4, Quartile 2 - 4; VE, vitamin E.

Model 1, adjusted for age, gender, and race;

Model 2, adjusted for factors in Model 1 plus education, cotinine, body mass index, ratio of family income to poverty, and physical activity;

Model 3, adjusted for factors in Model 2 plus hypertension and hypercholesteremia.

N = 3,692.

* $P < 0.05$.

Table S18. Multiple-adjusted linear regression between heavy metals and glucose in women aged 18 - 65.

		Continuous<br>β (95% CI)	Q1<br>(Reference)	Q2<br>β (95% CI)	Q3<br>β (95% CI)	Q4<br>β (95% CI)	P_{trend}
Ln concentrations of blood Pb (μg/dL), Cd, and Hg (μg/L)							
Ln-Pb	Model 1	0.11 (0.06, 0.16) *	1.00	0.07 (-0.01, 0.16)	0.11 (0.04, 0.18) *	0.24 (0.15, 0.32) *	< 0.01
	Model 2	0.12 (0.05, 0.19) *	1.00	0.05 (-0.04, 0.15)	0.10 (0.02, 0.17) *	0.27 (0.17, 0.37) *	< 0.01
	Model 3	0.09 (0.02, 0.16) *	1.00	0.05 (-0.04, 0.15)	0.05 (-0.04, 0.13)	0.23 (0.12, 0.34) *	< 0.01
Ln-Cd	Model 1	0.05 (0.02, 0.09) *	1.00	0.06 (-0.01, 0.13)	0.10 (0.01, 0.20) *	0.11 (0.04, 0.19) *	< 0.01
	Model 2	0.08 (0.02, 0.13) *	1.00	0.07 (-0.01, 0.14)	0.13 (0.03, 0.23) *	0.16 (0.04, 0.28) *	< 0.01
	Model 3	0.07 (0.01, 0.13) *	1.00	0.07 (-0.01, 0.15)	0.12 (0.02, 0.22) *	0.16 (0.02, 0.29) *	< 0.01
Ln-Hg	Model 1	0.01 (-0.01, 0.04)	1.00	-0.02 (-0.10, 0.06)	-0.04 (-0.11, 0.02)	0.03 (-0.04, 0.11)	0.50
	Model 2	0.05 (0.02, 0.07) *	1.00	-0.05 (-0.14, 0.05)	-0.06 (-0.14, 0.02)	0.10 (0.02, 0.18) *	0.03
	Model 3	0.04 (0.01, 0.06) *	1.00	-0.05 (-0.15, 0.06)	-0.08 (-0.17, 0.00)	0.06 (-0.03, 0.15)	0.30
Intake, mg/day							
VE	Model 1	-0.01 (-0.01, 0.00) *	1.00	-0.10 (-0.30, 0.10)	0.05 (-0.09, 0.20)	-0.06 (-0.20, 0.07)	0.83
	Model 2	-0.01 (-0.01, 0.01)	1.00	-0.07 (-0.22, 0.07)	0.05 (-0.14, 0.24)	-0.02 (-0.17, 0.14)	0.77
	Model 3	-0.01 (-0.01, -0.00) *	1.00	-0.10 (-0.26, 0.07)	0.03 (-0.18, 0.24)	-0.11 (-0.27, 0.05)	0.33

Note: Cd, cadmium; CI, confidence interval; Hg, mercury; Pb, lead; Q1, Quartile 1 (as reference); Q2 - 4, Quartile 2 - 4; VE, vitamin E.

Model 1, adjusted for age, gender, and race;

Model 2, adjusted for factors in Model 1 plus education, cotinine, body mass index, ratio of family income to poverty, and physical activity;

Model 3, adjusted for factors in Model 2 plus hypertension and hypercholesteremia.

N = 3,909.

* $P < 0.05$.

Table S19. Multiple-adjusted linear regression between heavy metals and glucose in participants aged 18 - 65 with BMI < 25 kg/m^2.

		Continuous<br>*β (*95% CI)	Q1<br>(Reference)	Q2<br>*β (*95% CI)	Q3<br>*β (*95% CI)	Q4<br>*β (*95% CI)	*P* trend
Ln concentrations of blood Pb (µg/dL), Cd, and Hg (µg/L)							
Ln-Pb	Model 1	0.10 (0.06, 0.15) *	1.00	0.06 (-0.03, 0.15)	0.12 (0.03, 0.21)	0.22 (0.13, 0.31) *	< 0.01
	Model 2	0.07 (0.01, 0.13) *	1.00	0.07 (-0.03, 0.18)	0.07 (-0.04, 0.18)	0.18 (0.06, 0.29) *	< 0.01
	Model 3	0.04 (-0.03, 0.11)	1.00	0.08 (-0.05, 0.20)	0.03 (-0.10, 0.16)	0.12 (-0.01, 0.25)	0.14
Ln-Cd	Model 1	0.04 (0.01, 0.07) *	1.00	0.03 (-0.06, 0.12)	0.10 (0.02, 0.19) *	0.07 (-0.02, 0.15)	0.05
	Model 2	0.03 (-0.03, 0.08)	1.00	0.01 (-0.09, 0.11)	0.08 (-0.02, 0.18)	0.01 (-0.12, 0.14)	0.39
	Model 3	0.03 (-0.03, 0.10)	1.00	0.07 (-0.05, 0.19)	0.12 (0.01, 0.24) *	0.02 (-0.14, 0.17)	0.32
Ln-Hg	Model 1	0.02 (-0.01, 0.05)	1.00	0.03 (-0.05, 0.12)	0.01 (-0.09, 0.09)	0.06 (-0.03, 0.15)	0.34
	Model 2	0.02 (-0.02, 0.06)	1.00	0.05 (-0.05, 0.15)	0.01 (-0.10, 0.11)	0.05 (-0.06, 0.16)	0.53
	Model 3	0.02 (-0.03, 0.06)	1.00	0.06 (-0.06, 0.19)	0.02 (-0.19, 0.14)	0.03 (-0.10, 0.15)	0.87
Intake, mg/day							
VE	Model 1	-0.00 (-0.01, 0.01)	1.00	-0.02 (-0.10, 0.07)	0.03 (-0.05, 0.12)	-0.03 (-0.12, 0.05)	0.73
	Model 2	-0.00 (-0.01, 0.01)	1.00	-0.02(-0.12, 0.08)	0.03 (-0.07, 0.14)	-0.03 (-0.14, 0.08)	0.82
	Model 3	-0.00 (-0.01, 0.01)	1.00	-0.06 (-0.18, 0.07)	0.02 (-0.11, 0.14)	-0.09 (-0.21, 0.03)	0.29

Note: Cd, cadmium; CI, confidence interval; Hg, mercury; Pb, lead; Q1, Quartile 1 (as reference); Q2 - 4, Quartile 2 - 4; VE, vitamin E.

Model 1, adjusted for age, gender, and race;

Model 2, adjusted for factors in Model 1 plus education, cotinine, body mass index, ratio of family income to poverty, and physical activity;

Model 3, adjusted for factors in Model 2 plus hypertension and hypercholesteremia.

N = 2,450.

* *P* < 0.05.

Table S20. Multiple-adjusted linear regression between heavy metals and glucose in participants aged 18 - 65 with BMI ≥ 25 kg/m^2.

		Continuous	Q1	Q2	Q3	Q4	P trend
		β (95% CI)	(Reference)	β (95% CI)	β (95% CI)	β (95% CI)	
Ln concentrations of blood Pb (μg/dL), Cd, and Hg (μg/L)							
Ln-Pb	Model 1	0.08 (0.03, 0.14) *	1.00	0.03 (-0.06, 0.11)	0.09 (0.01, 0.17) *	0.22 (0.12, 0.32) *	<0.01
	Model 2	0.07 (0.01, 0.13) *	1.00	0.06 (-0.04, 0.16)	0.13 (0.03, 0.22) *	0.22 (0.11, 0.34) *	<0.01
	Model 3	0.04 (-0.03, 0.11)	1.00	0.05 (-0.06, 0.15)	0.08 (-0.02, 0.19)	0.17 (0.04, 0.30) *	<0.01
Ln-Cd	Model 1	0.03 (-0.01, 0.06)	1.00	0.08 (-0.01, 0.17)	0.07 (-0.00, 0.14)	0.10 (0.03, 0.18) *	<0.01
	Model 2	0.05 (-0.01, 0.09)	1.00	0.10 (-0.01, 0.20)	0.07 (-0.01, 0.15)	0.12 (0.01, 0.23) *	0.03
	Model 3	0.04 (-0.01, 0.09)	1.00	0.13 (0.01, 0.24) *	0.08 (-0.01, 0.17)	0.12 (0.01, 0.24) *	0.04
Ln-Hg	Model 1	0.03 (0.01, 0.06) *	1.00	-0.05 (-0.14, 0.04)	-0.03 (-0.11, 0.06)	0.06 (-0.01, 0.13)	0.09
	Model 2	0.04 (0.01, 0.07) *	1.00	-0.06 (-0.16, 0.04)	-0.05 (-0.15, 0.05)	0.06 (-0.02, 0.14)	0.15
	Model 3	0.03 (-0.01, 0.06)	1.00	-0.075 (-0.18, 0.03)	-0.05 (-0.17, 0.06)	0.02 (-0.07, 0.12)	0.48
Intake, mg/day							
VE	Model 1	-0.01 (-0.01, 0.01)	1.00	-0.07 (-0.25, 0.11)	-0.04 (-0.18, 0.09)	-0.09 (-0.20, 0.03)	0.24
	Model 2	-0.00 (-0.01, 0.01)	1.00	-0.01 (-0.175, 0.16)	-0.03 (-0.19, 0.13)	-0.04 (-0.17, 0.10)	0.59
	Model 3	-0.00 (-0.01, 0.01)	1.00	-0.04 (-0.23, 0.14)	-0.10 (-0.27, 0.06)	-0.11 (-0.24, 0.03)	0.13

Note: Cd, cadmium; CI, confidence interval; Hg, mercury; Pb, lead; Q1, Quartile 1 (as reference); Q2 - 4, Quartile 2 - 4; VE, vitamin E.

Model 1, adjusted for age, gender, and race;

Model 2, adjusted for factors in Model 1 plus education, cotinine, body mass index, ratio of family income to poverty, and physical activity;

Model 3, adjusted for factors in Model 2 plus hypertension and hypercholesteremia.

$N = 5{,}084$.

* $P < 0.05$.

Table S21. Associations of blood heavy metals with diabetes and glucose by VE intake levels with Model 2 adjustment in participants aged 18 - 65.#

Metal quartiles by VE levels	Diabetes			Glucose					
	Ln-Pb			Ln-Pb		Ln-Cd		Ln-Hg	
	Case/participants	%	OR (95% CI)	Glucose (mmol/L)	β (95% CI)	Glucose (mmol/L)	β (95% CI)	Glucose (mmol/L)	β (95% CI)
**Level 1**									
Q1	15/466	3.22	1.00 (reference)	5.47	1.00 (reference)	5.58	1.00 (reference)	5.55	1.00 (reference)
Q2	18/480	3.75	12.15 (2.24, 65.91) *	5.50	0.16 (-0.11, 0.43)	5.60	0.12 (-0.11, 0.36)	5.59	0.10 (-0.07, 0.28)
Q3	32/469	6.82	3.46 (0.53, 22.49)	5.61	0.19 (0.02, 0.35) *	5.58	0.12 (-0.12, 0.37)	5.56	0.01 (-0.26, 0.28)
Q4	37/483	7.66	3.36 (0.84, 13.30)	5.76	0.25 (0.05, 0.44) *	5.59	0.02 (-0.24, 0.29)	5.64	0.06 (-0.10, 0.24)
**Level 2**									
Q1	33/834	3.96	1.00 (reference)	5.40	1.00 (reference)	5.59	1.00 (reference)	5.57	1.00 (reference)
Q2	37/1049	3.53	0.99 (0.26, 3.69)	5.49	0.11 (-0.00, 0.23)	5.58	0.04 (-0.11, 0.21)	5.55	0.15 (-0.03, 0.33)
Q3	48/950	5.05	4.51 (0.93, 21.88)	5.65	0.17 (0.02, 0.33) *	5.54	0.05 (-0.13, 0.25)	5.58	0.22 (0.06, 0.38) *
Q4	73/961	7.60	7.25 (1.79, 29.39) *	5.77	0.36 (0.14, 0.57) *	5.61	0.05 (-0.17, 0.27)	5.61	0.12 (-0.06, 0.31)
**Level 3**									
Q1	12/405	2.96	1.00 (reference)	5.39	1.00 (reference)	5.57	1.00 (reference)	5.55	1.00 (reference)
Q2	26/536	4.85	1.22 (0.35, 4.23)	5.54	0.10 (-0.08, 0.29)	5.56	-0.11 (-0.32, 0.10)	5.53	-0.05 (-0.24, 0.13)
Q3	27/470	5.74	0.70 (0.18, 2.67)	5.63	0.20 (0.02, 0.37) *	5.64	-0.14 (-0.30, 0.02)	5.59	0.02 (-0.18, 0.23)
Q4	29/497	5.84	1.40 (0.37, 5.24)	5.67	0.13 (-0.04, 0.31)	5.48	0.06 (-0.17, 0.30)	5.59	0.03 (-0.18, 0.25)

Note: Cd, cadmium; CI, confidence interval; Hg, mercury; OR, odds ratio; Pb, lead; Q1, Quartile 1 (as reference); Q2 - 4, Quartile 2 - 4; VE, vitamin E.

# The model was adjusted for age, gender, race, education, cotinine, body mass index, ratio of family income to poverty, and physical activity. $N = 7{,}601$.

* $P < 0.05$.

Table S22. Associations of blood heavy metals with diabetes and glucose by VE intake levels with Model 3 adjustment in participants aged 18 - 65.#

Metal quartiles by VE levels	Diabetes			Glucose					
	Ln-Pb			Ln-Pb		Ln-Cd		Ln-Hg	
	Case/participants	%	OR (95% CI)	Glucose (mmol/L)	β (95% CI)	Glucose (mmol/L)	β (95% CI)	Glucose (mmol/L)	β (95% CI)
**Level 1**									
Q1	15/466	3.22	1.00 (reference)	5.47	1.00 (reference)	5.58	1.00 (reference)	5.55	1.00 (reference)
Q2	18/480	3.75	17.74 (2.40, 131.13) *	5.50	0.24 (-0.09, 0.57)	5.60	0.05 (-0.21, 0.33)	5.59	0.08 (-0.15, 0.31)
Q3	32/469	6.82	2.72 (0.23, 31.60)	5.61	0.14 (-0.07, 0.35)	5.58	0.09 (-0.20, 0.39)	5.56	-0.00 (-0.34, 0.34)
Q4	37/483	7.66	3.21 (0.48, 21.32)	5.76	0.22 (-0.01, 0.46)	5.59	0.01 (-0.29, 0.32)	5.64	0.01 (-0.19, 0.23)
**Level 2**									
Q1	33/834	3.96	1.00 (reference)	5.40	1.00 (reference)	5.59	1.00 (reference)	5.57	1.00 (reference)
Q2	37/1049	3.53	0.72 (0.19, 2.80)	5.49	0.05 (-0.08, 0.18)	5.58	0.07 (-0.09, 0.23)	5.55	0.17 (-0.03, 0.38)
Q3	48/950	5.05	3.05 (0.81, 11.49)	5.65	0.11 (-0.05, 0.28)	5.54	0.08 (-0.11, 0.29)	5.58	0.19 (0.02, 0.37) *
Q4	73/961	7.60	4.98 (1.08, 22.90) *	5.77	0.25 (0.01, 0.48) *	5.61	0.05 (-0.17, 0.29)	5.61	0.07 (-0.11, 0.26)
**Level 3**									
Q1	12/405	2.96	1.00 (reference)	5.39	1.00 (reference)	5.57	1.00 (reference)	5.55	1.00 (reference)
Q2	26/536	4.85	1.10 (0.28, 4.29)	5.54	0.14 (-0.06, 0.36)	5.56	-0.14 (-0.35, 0.06)	5.53	-0.08 (-0.31, 0.15)
Q3	27/470	5.74	0.33 (0.07, 1.47)	5.63	0.19 (0.00, 0.37) *	5.64	-0.18 (-0.36, 0.00)	5.59	0.02 (-0.20, 0.26)
Q4	29/497	5.84	0.93 (0.22, 3.98)	5.67	0.08 (-0.11, 0.28)	5.48	0.04 (-0.20, 0.28)	5.59	0.02 (-0.20, 0.25)

Note: Cd, cadmium; CI, confidence interval; Hg, mercury; OR, odds ratio; Pb, lead; Q1, Quartile 1 (as the reference group); Q2 - 4, Quartile 2 - 4; VE, vitamin E.

# The model was adjusted for age, gender, race, education, cotinine, body mass index, ratio of family income to poverty, physical activity, hypertension, and hypercholesteremia. $N = 7{,}601$.

* $P < 0.05$.
